# Multisensory temporal binding induces an illusory gap/overlap that reduces the expected audiovisual interactions on saccades but not manual responses

**DOI:** 10.1371/journal.pone.0266468

**Published:** 2022-04-07

**Authors:** Manuel Vidal, Françoise Vitu

**Affiliations:** 1 Institut de Neurosciences de la Timone, UMR 7289, CNRS, Aix-Marseille Université, France; 2 Laboratoire de Psychologie Cognitive, UMR 7290, CNRS, Aix-Marseille Université, France; Tokai University, JAPAN

## Abstract

Throughout the day, humans react to multisensory events conveying both visual and auditory signals by rapidly reorienting their gaze. Several studies showed that sounds can impact the latency of visually guided saccades depending on when and where they are delivered. We found that unlocalized beeps delivered near the onset time of a visual target reduce latencies, more for early beeps and less for late beeps, however, this modulation is far weaker than for perceptual temporal judgments. Here we tested our previous assumption that beeps shift the perceived timing of target onset and result in two competing effects on saccade latencies: a multisensory modulation in line with the expected perceptual effect and an illusory gap/overlap effect, resulting from target appearance being perceived later/closer in time than fixation offset and shortening/lengthening saccade latencies. Gap/overlap effects involve an oculomotor component associated with neuronal activity in the superior colliculus (SC), a multisensory subcortical structure devoted to sensory-motor transformation. We therefore predicted that the interfering illusory gap/overlap effect would be weaker for manual responses, which involve distinct multisensory areas. In three experiments we manipulated the delay between target onset and an irrelevant auditory beep (stimulus onset asynchrony; SOA) and between target onset and fixation offset (real gap/overlap). Targets appeared left/right of fixation and participants were instructed to make quick saccades or button presses towards the targets. Adding a real overlap/gap (50% of SOA) compensated for the illusory gap/overlap by increasing the beep-related modulation of saccade latencies across the entire SOA range, whereas it barely affected manual responses. However, although auditory and gap/overlap effects modulated saccade latencies in similar ways, these were additive and could saturate, suggesting that they reflect independent mechanisms. Therefore, multisensory temporal binding affects perception and oculomotor control differently, likely due to the implication of the SC in saccade programming and multisensory integration.

## Introduction

Humans and other mammals have evolved to react quickly to sudden events in their environment, combining signals provided by different senses to produce an appropriate behavior. This is the case for instance when a mosquito tries to bite our legs. Once the insect lands on one of our thighs, two non-exclusive eye and hand behavioral reactions may be observed: either we presume that the blurred spot in peripheral vision is a mosquito and we immediately attempt to slap it with our hand, or we perform a quick saccade to place the blurred spot onto the central part of the retina–the fovea–where enhanced visual acuity allows identifying the insect. The problem yet is that nasty mosquitoes often do not fly silently, but produce an irritating sound that we can hear as they get closer. This auditory signal is known to combine with the visual image and possibly alter the perceived timing of the multimodal event, the buzzing mosquito. The present study aims at characterizing differences in the level of interference caused by auditory signals on ocular and manual reactions to visual events, considering the time course of activation of the underlying neural networks.

### Multisensory temporal binding and saccades

Most events of our everyday life are intrinsically multisensory. Clapping hands, talking people, or objects hitting the ground produce both acoustic and visual signals that are transmitted in the air and transduced by our sensory systems at different speeds. Although these signals reach the multisensory areas where they are associated at different times, depending notably on the distance from the source, they usually combine into a single multisensory event to be perceived as simultaneous [1–3]. When a flash is presented just before or after a short beep, the visual stimulus is perceived closer in time to the auditory stimulus than it actually is [4]. Consistently, the perception of a flash shifts forward/backward in time when paired with a lagging/leading sound click [5]. This temporal ventriloquism also has consequences in visual tasks involving saccadic eye movements. The execution of a saccade tends to bias the perceived location of a flash displayed shortly before or after the eyes move. This localization error was shown to vary with the addition of a tone, as if the flash had occurred closer in time to the tone, when the delay between the flash and the tone was short enough for the two modalities to be bound together [6]. It yet remains unclear whether and how multisensory temporal binding influences the generation/planning of visually guided saccades.

Saccades are programmed in the superior colliculus (SC), an integrative and multilayered midbrain structure devoted to sensory-motor transformation [7–9]. The SC is also the first-identified multisensory brain structure where audiovisual integration in time and space takes place [10–12]. Neurons in the superficial layers of the SC respond exclusively to visual stimuli and neurons in the intermediate and deeper layers, where the spatial code for a saccade is computed, respond to both visual and auditory stimuli, such that auditory and visual sensory maps are connected at a very early processing stage [13]. Audiovisual interactions in monkeys were found to modulate saccade-related activity in the SC, and in turn to affect saccade latency [14]. Relatedly, several behavioral studies in humans reported an influence of non-visual signals on the generation of visually-guided saccades, showing in particular that auditory stimulations affect saccade latency, even when they are irrelevant for the task [15–20]. In these studies, temporal binding between the visual target and the auditory stimulation could be responsible for the effect on saccade latencies, as it was reported by Maij et al. for a visual-localization task in a saccade-targeting paradigm [6].

In our previous work [20], we characterized the influence of auditory beeps on the generation of saccades towards single peripheral visual targets, by distinguishing two effects. The first, referred to as *warning effect*, corresponded to a global (11 ms) decrease in the mean latency of saccades when the beep and the target were delivered together (SOA = 0 ms). The second, referred to as *modulation effect*, corresponded to a non-linear modulation of saccadic reaction times (SRTs) depending on the timing of the beep, with shorter–or longer–mean latencies for beeps delivered before–or after–target onset. For beeps occurring after the saccade (SOA = 240 ms), latencies returned back to the no-beep baseline. The warning effect could result from increased alertness, the external sound acting as a warning signal announcing the upcoming visual target, and even more so as auditory information is processed faster compared to visual information [19, 21]. Another possible interpretation is that the beep captured attention, and favored an early fixation disengagement [17, see also 22]. For the modulation effect, we attributed it to the perceived timing of the visual event, which was likely shifted towards the irrelevant auditory beep [3], and subsequently influenced the detection and the coding of the target in the deep layers of SC. However, within the tested range of SOAs, the size of the modulation effect (about 22ms in [20]) was far weaker than what could be expected based on purely perceptual temporal judgments (about 120 ms in [3]) involving multisensory cortical areas. Our assumption, as further detailed below, is that this was a direct consequence of audiovisual temporal binding creating an illusory gap/overlap paradigm, and limiting in turn the effect of an irrelevant sound on the latency of visually guided saccades.

### A possible illusory gap/overlap induced by multisensory temporal binding

**Fig 1:**
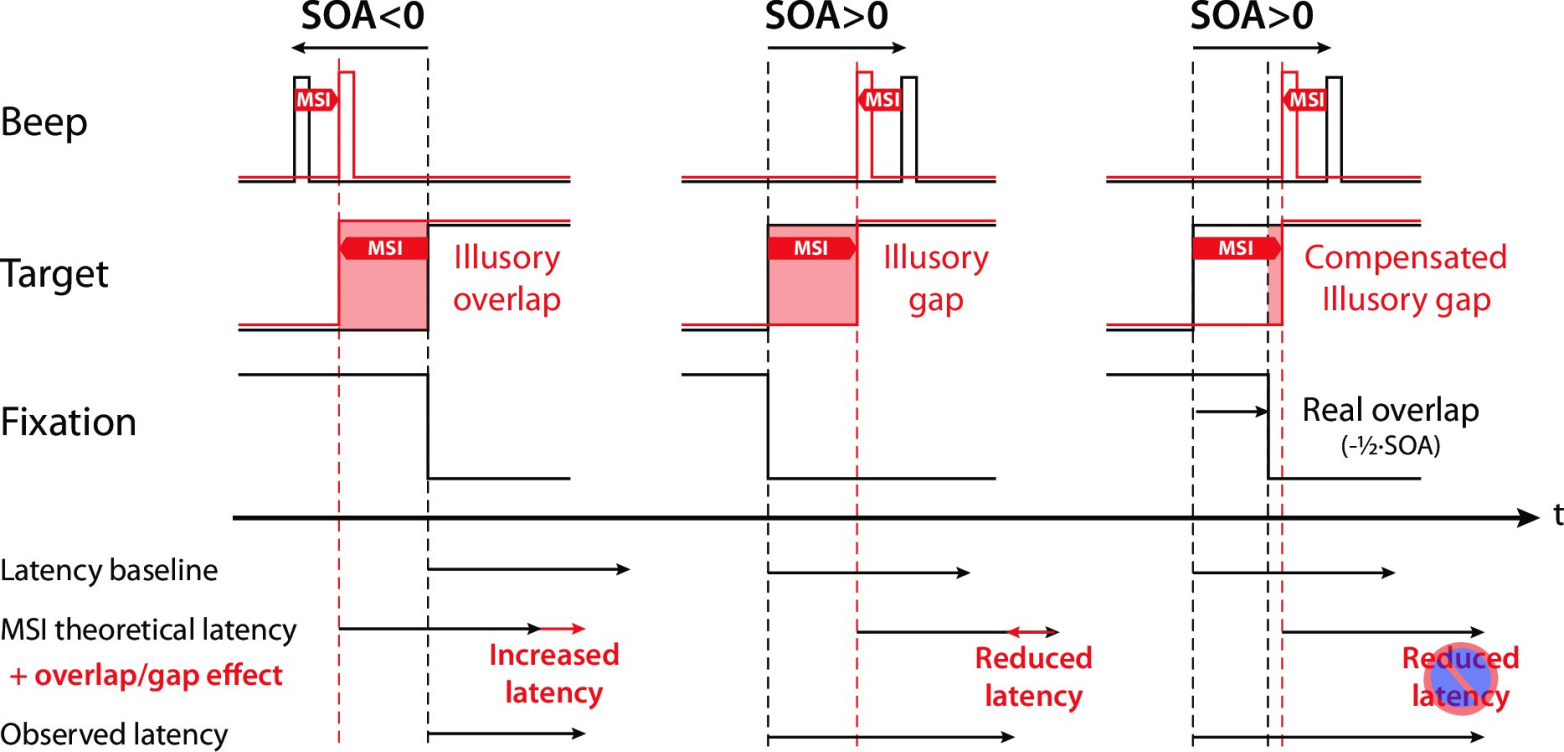
Illusory gap/overlap in the construction of saccade latencies. Illustration of how multisensory integration (MSI) could introduce an illusory overlap/gap for negative/positive SOAs, by binding temporally the target onset with the beep signal (left/middle panel). This shift in time of the perceived target onset would produce an MSI theoretical latency that also shifts. However, the illusory overlap/gap would also produce an increase/decrease of saccade latencies that would go against the MSI theoretical effect and reduce the observed latency modulation. The right panel illustrates how adding a real overlap, arbitrarily set to half of the SOA, could compensate for the illusory gap introduced by the MSI for positive SOAs and thereby allowing a larger expression of the MSI modulation in the observed saccade latency.

It has long been established, since Saslow’s original work [23], that the latency of saccades towards suddenly appearing peripheral visual targets greatly varies depending on the relative timing of target onset and fixation-point offset [22] (for a review see [24]). When the fixation stimulus remains on (for some time) after target onset as in an overlap paradigm, saccade latency is inflated compared to a step condition, where the fixation stimulus is turned off precisely when the target appears. Reversely, when the fixation stimulus is extinguished some delay before target onset, as in a gap paradigm, saccade latency is reduced compared to the step baseline, while a separate population of early-triggered, express, saccades sometimes emerges [22]. Express saccades were reported in several studies. However, they seem to emerge in rather specific conditions, e.g., low uncertainty of target location, and extensive training, and were reported essentially in monkeys [for a review see 25]. The possibility that they are purely anticipatory responses has been raised [26]. Given the laws of multisensory temporal integration [3–5], similar effects likely modulate the impact of irrelevant auditory beeps on the generation of visually guided saccades. In our previous study for instance, we used a step paradigm, meaning that the offset of the fixation stimulus was simultaneous with the display of the visual target [20]. Still, the perceived timing of target onset likely shifted towards the additional auditory signal [3], hence resulting in display conditions that were perceptually similar to a gap or an overlap paradigm depending on the sign of the SOA. When the beep was delivered before the visual target (negative SOAs), the target seemed to appear earlier than it truly appeared, and before fixation offset, thus producing an illusory overlap condition (left panel of **Fig 1**). Conversely, when a beep was delivered after the target (positive SOAs), the perceived timing of target onset was delayed, leading to an illusory gap (middle panel of **Fig 1**). Consistent with this assumption is the fact that during debriefing, some of our participants reported a peculiar temporal sequencing of target onset and fixation offset when the beep occurred after target onset: although both events were always perfectly synchronous (step paradigm), it seemed as if, for a brief instant, there was neither fixation nor target on the screen. In other words, it felt as if a temporal gap had been introduced between the two visual events. Assuming such illusory gap/overlap conditions reduce/increase saccadic latency just like veridical gap/overlap conditions do, compared to a step condition, it appears that audiovisual temporal binding could produce an effect on saccadic latency just opposite to what would be expected purely based on multisensory interactions, hence countering the influence an irrelevant auditory stimulus has on visually guided saccades. Whereas the illusory gap associated with late beeps (positive SOAs) would reduce the beep-related increase in saccade latency, the illusory overlap associated with early beeps (negative SOAs) would at least partially cancel the beep-related decrease of saccade latency. It yet remains difficult to figure out whether these effects would simply be additive. Indeed, gap/overlap effects rely on mechanisms that might overlap, even just partially, with those responsible for audio-visual interactions.

The gap effect, just like the effect of an auditory stimulus preceding (or simultaneous with) the onset of a visual target, is commonly assumed to involve a first, non-specific, warning-type component [19, 24]. The underlying hypothesis is that the pre-target fixation-stimulus extinction signals the impending target appearance and in turn allows to be temporally prepared for a (saccadic) response, in line with the observation of a gap effect also with manual responses [19, 27, but see 28]. A foveal offset yet is a specific sensory event for the oculomotor system that does more than simply raising alertness. When its warning effect is neutralized using a warning tone preceding target onset in both gap and overlap conditions, it still shortens the latency of visually guided saccades, but no longer affects manual reaction times (MRTs) [29]. Moreover, unlike an auditory offset which influences SRTs as much as an auditory onset, a foveal visual offset affects saccade latency like no other visual event, while non-differentially affecting manual responses [19, 27, see also 30]. When it occurs before target appearance, it reduces saccade latency to a greater extent than either a central visual onset or a peripheral visual offset before the target. Conversely, when it follows target onset, it no longer affects saccade initiation, unlike a visual onset, and even more so a foveal onset, that delays saccade-onset time whether it occurs after or simultaneously with the target (the remote distractor effect, see also [31, 32]; for manual responses see [33, 34]). Such a continuum of reciprocal offset/onset effects on saccades, but not manual responses, led to the proposal that another component, specific to visuo-motor orienting responses, contributes to the gap effect. The general assumption, in terms of a fixation system, is that the physical disappearance of the fixation stimulus before or simultaneously with the saccade target expedites fixation release, and possibly also attentional disengagement, thereby speeding-up the initiation of a saccade [19, 24, 27, see also 22]. This would take place in the SC, where saccades are programmed, as further assessed by the dynamics of visually based neuronal activity in monkeys’ SC during the gap period: a progressive reduction in the activity of rostrally located neurons, coding for the fovea [35, 36] and projecting massively onto downstream omnipause neurons involved in fixation behavior [37], and a reciprocal rise in the discharge rate of buildup neurons associated with large-amplitude saccades [38]. Such a reciprocal activity pattern may additionally favor the buildup of spatially localized, saccade-related, activity during the gap period [39], and even more so as the predictability of target location increases [40], thus suggesting, in line with several behavioral findings, that advanced motor preparation may also contribute to the gap effect [25, 29, 41, 42].

The SC is known to drive a range of orienting responses besides eye movements, including arm, trunk and head movements in coordination with orientation gaze shifts [43, 44]. The SC, however, does not control manual key presses, which mostly involve primary and supplementary motor areas. This explains both why there is no offset-specific component to the gap effect observed with manual responses, but only a general warning-type component, and why a robust gap effect could be observed for hand pointing [45, 46], that was much stronger than for choice manual responses [45, 47]. Importantly though, the SC is an integrative structure that receives direct visual input from the retina and the primary visual cortex, as well as afferent projections from other cortical areas, including the frontal eye fields (FEFs), which are involved in selection and decision processes, as well as voluntary fixation disengagement and prediction [48]. The warning signal associated with fixation offset, as with any other pre-target sensory event, would therefore also contribute to fixation disengagement, but through top-down projections to the SC rather than more direct visual pathways, which likely underlie the offset-specific component of the gap effect [24]. The fact that the gap effect minus its warning component was found to be greater for reflexive pro-saccades compared to anti-saccades, which necessarily involve the FEF, corroborates this view [21, 29]. The SC also integrates afferents from cortical and subcortical areas involved in auditory processing [49], thus suggesting that an irrelevant auditory stimulus has the potential to directly modulate neuronal activity in the SC. Audiovisual interactions in the SC yet are complex, depending notably on the alignment in space and time of visual and auditory signals [10, 17], thus making it difficult to predict whether a beep, and even more so an unlocalized (head-centered) sound, would modulate the fixation-move balance, and would potentially interact with an illusory gap/overlap induced by multisensory temporal binding.

### Rationale

In line with several previous findings, we recently reported a modulation in the latency of visually guided saccades by irrelevant auditory beeps, that was much reduced compared to what could be expected based on multisensory laws. We proposed the hypothesis that an illusory gap/overlap–related to beeps shifting the perceived onset time of the target–could have minimized the effect of beeps on saccades. Here we put this hypothesis to a strong test. In a first experiment, we introduced a physical overlap/gap to compensate for the potential illusory gap/overlap and tested whether and how this alters the impact of audiovisual interactions on saccades as well as manual responses (Experiment 1a and 1b). Under the assumption that an illusory gap/overlap acts on saccades like real ones, and only has limited influence on manual responses as previously reported, we can draw two predictions. First, within the same SOA range, we should observe a larger beep-related modulation effect of latencies for manual responses compared to saccades. Second, since distinct cerebral regions are involved in motor and oculomotor tasks, we would expect that the addition of a physical overlap/gap would increase the modulation effect on SRTs (right panel of **Fig 1**) but leave MRTs unaffected. In the next two experiments, we then tested whether gap/overlap and multisensory effects on saccade latency rely on independent mechanisms, and whether they are additive and can saturate (Experiments 2 and 3).

## Materials and methods

### Participants

In all experiments, participants were either students or researchers from the university; they were all naïve concerning the purpose of the experiment except for the first author. Participants were selected on a voluntary basis and student participants received a financial compensation of 10€/hour. They all gave their prior written consent after being informed of the methods used and their right to interrupt the experiment at any time. This project was approved by the *Comité d’éthique d’Aix-Marseille Université* (reference 2014-12-3-06) and complies with the regulations described in the Declaration of Helsinki (2012).

### Apparatus and stimuli

Participants sat in front of a computer screen with their head movements being restricted by a chin and head rest. Stimuli were generated on a PC computer running Windows 7 operating system. Routines were written in Matlab 9.3.0 using the PsychToolbox 3.0.9 [50, 51]. The right eye position was recorded using an SR Research EyeLink 1000 video eye tracker (sampling at 1000Hz) mounted on the same structure as the chin rest. Visual stimuli were displayed on a Sony Trinitron CRT monitor running at a resolution of 1024×768 and a refresh rate of 100Hz (frame duration of 10 ms). The chin rest was adjusted so that the participants’ eyes, when in their primary position, were aligned with the center of the screen, at a distance of 57 cm. The fixation point was a small white disk (0.12° in diameter) displayed at the center of the screen. The visual target was a white disk (0.36° in diameter) that could appear either to the left or to the right of the fixation stimulus at an eccentricity of 8°. Background was set to 50% grey level (25.8 cd/m^2^ luminance after gamma correction). Beeps were 20 ms 880 Hz tones attenuated by a raised-cosine waveform (50% at 10 ms) delivered binaurally through Beyerdynamics DT770 closed headphones. The computer audio driver was set so that the audiovisual jitter remained below 1 ms. Timing accuracy of visual and auditory stimuli was controlled using a dual-channel oscilloscope connected to both the auditory output and a photosensitive cell placed directly on the screen.

### Procedure

**Fig 2:**
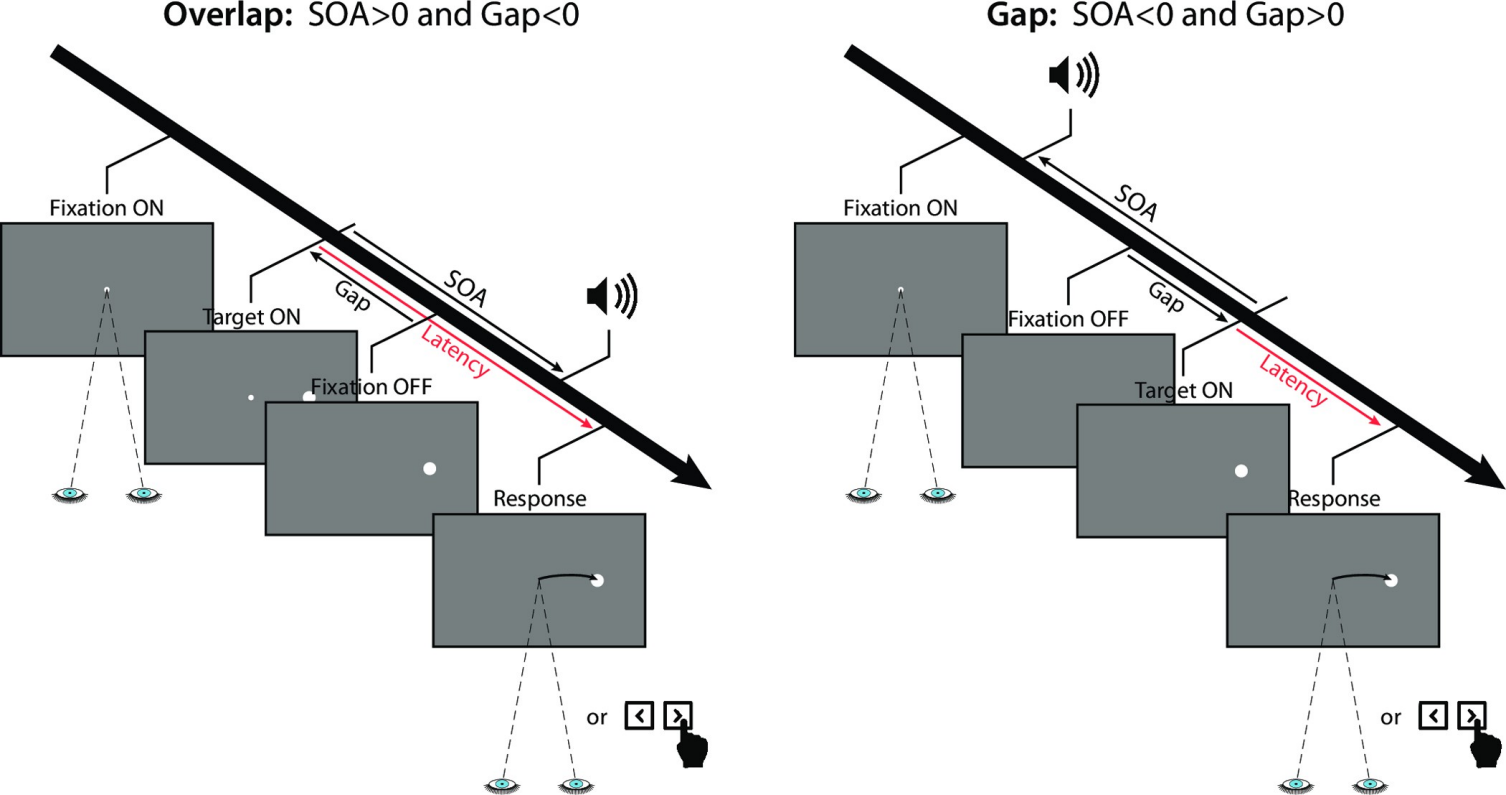
Time course of a trial illustrating the overlap (left) and gap (right) situations, the beep signal and the response mode for the experiments. A central fixation spot was presented for a random duration ranging from 750 to 1250 ms. The target appeared left/right at an eccentricity of 8°, either simultaneously (no-gap or step conditions), before (overlap conditions) or after the fixation offset (gap conditions). A short beep was played either before or after target onset (SOA ranging from −120 to +120 ms). In baseline conditions, no beep was played. Participants were instructed to shift their gaze towards the target (Experiments 1a, 2 and 3) or to indicate the target side by pressing the left/right mouse button (Experiment 1b), as fast and as accurately as possible. The response latency corresponded to the delay between target onset and the start of the saccade or the mouse-button press.

**Fig 2** illustrates the time course of events in a trial with the various factors manipulated in the three experiments. Trials started with a fixation point appearing in the center of the screen. Participants were asked to fixate it and to avoid blinking during the entire stimulus presentation. After a random delay that ranged from 750 ms to 1250 ms, the fixation stimulus was turned off. The visual target appeared left/right at an eccentricity of 8°, either before/after fixation offset (*Gap compensated* conditions in Experiments 1a, 1b and 2; *Gap conditions* in Experiment 3) or simultaneously with fixation offset (*Beep conditions* in Experiment 3). In most trials, a short beep was played around the time of target onset (SOA ranging from −120 to +120 ms). In the *No beep* baseline condition (Experiments 1a, 1b and 2) or in the *Gap conditions* (Experiment 3), no beep was played. Participants were instructed to shift their gaze towards the target (Experiments 1a, 2 and 3), or to indicate the side where the target appeared by pressing the left/right mouse button (Experiment 1b), as quickly and as accurately as possible. In order to reduce the occurrence of anticipatory responses, as in our previous study [20] participants were informed that the beeps conveyed no information about when or where the target would appear and were asked to ignore them. The response latency corresponded to the delay measured between the onset of the target and the beginning of the saccade or the mouse-button press.

### Data processing

In Experiments 1a, 2 and 3 where the motor responses were saccades, we used the Eyelink real-time engine to detect saccades and compute SRTs, setting velocity and acceleration thresholds to 30°/s and 8000°/s^2^ [52]. Trials in which no saccade were detected, or there was an eye blink, or saccades started too late (latency > 400 ms) were discarded. In Experiment 1b, where motor responses were button presses, MRTs were measured. The majority of anticipatory saccadic and manual responses were eliminated using an adaptive low-pass filter applied to the latency distributions for each participant and condition. Each cutoff threshold was determined taking the lowest value in the range of 80 to 120 ms (160 to 240 ms) sampled every 1 ms (2 ms) for saccades (manual responses) that left fewer than 1% of responses in the direction opposite to the target (e.g. left when the target appeared right). If 1% could not be reached, the cutoff threshold corresponding to the minimum was used. Because anticipatory responses have 50% chances to go in the opposite direction, limiting these below 1% mechanically limited anticipatory responses going in the right direction below 1%. This filter adapts to individual specificities by finding the optimal tradeoff between preserving a maximum of the visually-driven responses and removing most of the responses programmed before target onset/processing. Finally, responses in the opposite direction (in all experiments) and saccades falling short of the target (gain < 0.45 in Experiments 1a, 2 and 3) were excluded.

For each subject and each SOA condition, the median value and the median absolute deviation (MAD) of the response latency distribution were computed. To reduce inter-individual dispersion in statistical analyses and allow comparisons between experiments, we computed the corresponding normalized median latency and MAD according to the *No beep* condition baseline, as follows:

$$n_{score}(SOA)=\frac{x(SOA)-x(No\;beep)}{\sigma_{x}}$$ (1)

where *x* is either the median latency or the MAD of a given condition, and *σ*_*x*_ is the standard deviation of *x* across all conditions. For all experiments, the statistical analysis started with a two-way repeated measure ANOVA performed on the n_scores_ with Target direction as a two-level factor. In all experiments, there was neither a main effect of Target direction nor an interaction of Target direction with the other factors (SOA x Gap factor in Experiments 1a, 1b and 2; SOA x [Beep or Gap] in Experiment 3). Therefore, left and right responses were pooled together in a single distribution and the median of this collapsed distribution was further processed using ANOVAs and Student t-tests for planned comparisons. We previously checked that n_scores_ did not deviate from normality and that they met the homoscedasticity assumption required for parametric statistics, conducting a Shapiro-Wilk’s test and a Levene’s test. Except otherwise stated, all sets of measures had distributions that did not differ from normality, and compared sets had homogeneous variances (see **S1** and **S2 Tables**). The alpha value for significance was always corrected using Bonferroni’s rule depending on the number of comparisons performed on the same data set. Raw data files, global plots, individual plots and data processing code for all experiments can be found in the following public repository:

*https*:*//amubox*.*univ-amu*.*fr/s/dmYc5p8zbwpKjYp*

## Experiments 1a and 1b: Compensating for the Illusory gap/overlap

We previously proposed that the combined presentation of a visual target and an irrelevant beep shifts the perceived timing of the target, and introduces in turn an illusory gap/overlap, that minimizes the impact of the beep on saccade latencies [20]. We further hypothesized that such a deleterious effect may not generalize to manual responses, due to the much weaker (though less studied) influence of gaps/overlaps on manual compared to saccadic responses, as well as distinct underlying neural pathways. The goal of our first experiment was to test this assumption, that audiovisual temporal integration creates an illusory gap/overlap that specifically affects oculomotor responses. We first tested in Experiment 1a, whether the introduction of a physical gap/overlap between the fixation offset and the visual target onset affects the beep-related modulation of saccade latencies over a range of SOAs. In Experiment 1b, we replaced the ocular response by a manual keypress response. This allowed us to test, over the same SOA range, whether the beep-related modulation effect in the absence of a physical gap/overlap is larger for manual responses than for saccades, and whether the addition of a physical overlap/gap conversely has a weaker impact on the modulation effect for MRTs.

The real overlap/gap was set to half of the SOA to compensate–at least partially–for the illusory gap/overlap induced by the temporal binding between the visual target and the beep at positive/negative SOAs (right panel of **Fig 1**). This was determined completely arbitrarily, because our prior attempt to measure the illusory gap experimentally for each participant and each condition, using a temporal-order judgment task, remained unsuccessful: the task was extremely difficult and estimates were highly variable. Part of the multisensory effects found for saccades involve a neural circuitry–the SC and FEF–that is specific to oculomotor control. For manual responses, they essentially involve premotor and motor cortices that are fed with audiovisual signals produced in other multisensory areas–posterior superior temporal sulcus (pSTS). Moreover, the literature points to a limited gap/overlap effect on manual responses compared to goal directed saccades, likely due to an oculomotor-specific component associated with SC mechanisms. We therefore expected to obtain quantitatively different results depending on the response modality, with a potentially larger beep-related modulation effect for manual responses than for ocular responses in the absence of a physical gap/overlap. In addition, under the assumption that illusory gaps/overlaps act on saccades like real ones do, we expected the physical overlap/gap manipulation to increase the beep-related modulation effect on saccade latencies, but to leave that effect for manual responses essentially unaffected.

Twelve volunteers (nine women and three men) participated in both experiments. The order between the two experiments was counterbalanced across participants and for each participant the experiments were conducted on two different sessions separated by a minimum of two days. Participants were between 19 and 42 years old (average 25.4) and all were right-handed. Trial conditions were defined as the combination of three factors: [2 Gap factors (*No gap* and *Gap compensated*) × 5 SOAs (−120, −60, 0, +60, +120 ms) + *No beep* baseline] × 2 Target sides (left and right). In the compensated illusory gap conditions, we added a delay corresponding to -½ of the SOA between target onset and fixation offset. For instance, in conditions with a 120 ms positive SOA–beeps delivered 120 ms after target onset–we introduced a 60 ms negative gap corresponding to a 60 ms overlap between fixation offset and target onset. The task was to respond as quickly as possible to target onset, either by executing a saccade towards the target (Experiment 1a), or by pressing the left or right mouse button depending on the side where the target was displayed (Experiment 1b). Participants completed a total of four sessions of 240 trials each totalizing 960 trials (48 per Gap factor × SOA × Direction). The order of the conditions was randomized within blocks of 60 trials. Between each session, participants had a few minutes break to rest. After each block of 60 trials, participants could rest for a few seconds before resuming the experiment.

### Results

#### Effect on saccades (Experiment 1a)

**Fig 3:**
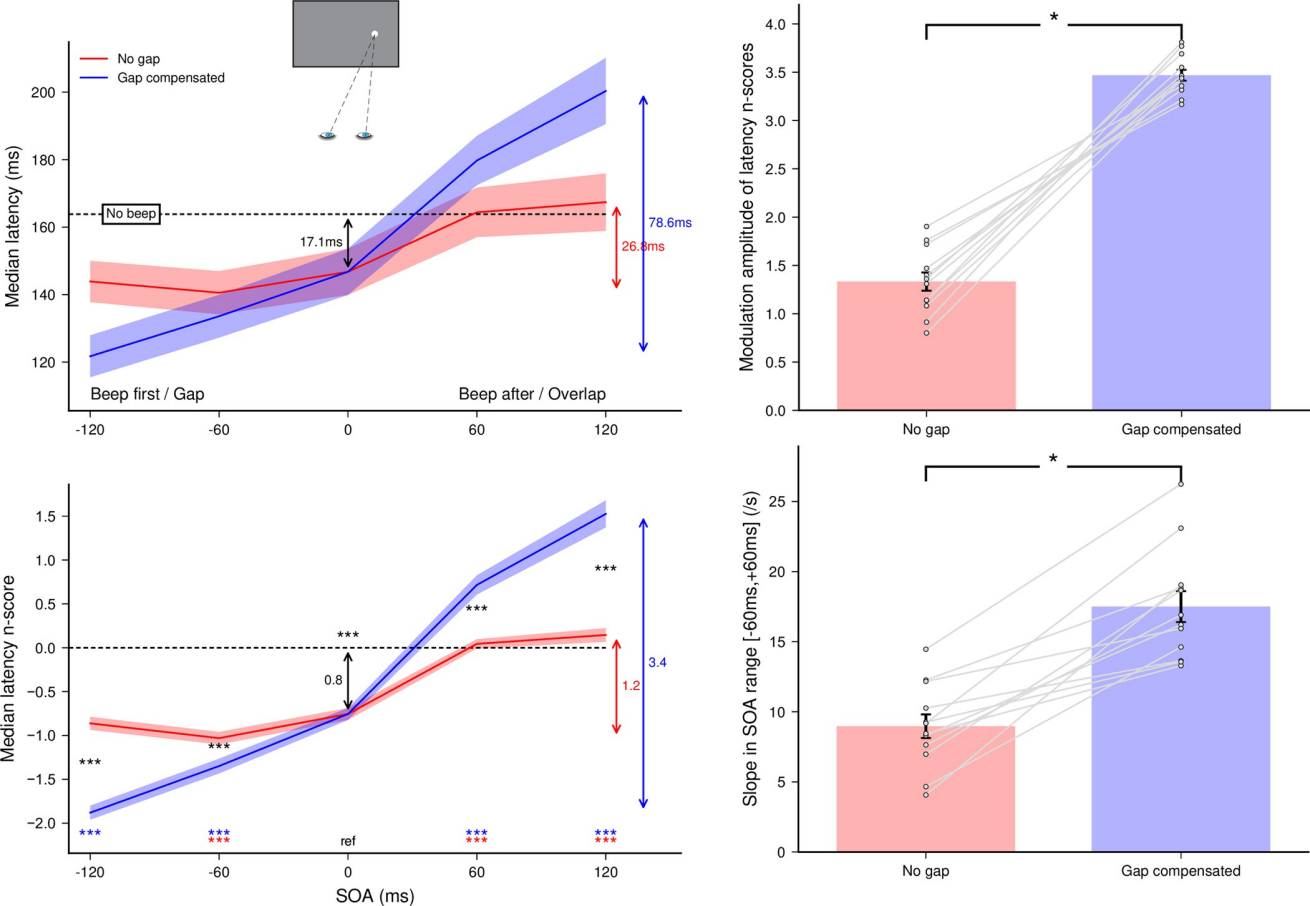
Experiment 1a. Effect of gap compensation on saccade latency. **(Left panel)** Median latencies averaged across participants for the *No gap* and *Gap compensated* conditions as a function of SOA (top) and their corresponding n_scores_ (bottom). Dashed lines show the *No beep* baseline condition level. Statistics performed on the n_scores_ included a single sample t-test to compare the 0-ms SOA reference condition with the *No beep* condition (black arrow), and for each gap factor separately, paired t-tests compared this reference with each SOA condition (colored stars for each SOA above the X-axis). Finally, paired t-tests compared *No gap* and *Gap compensated* conditions for each SOA (black stars between curves). Three stars indicate highly significant differences for the paired t-tests after Bonferroni correction (p<0.00556). **(Right panel)** Modulation amplitude of latency median n_scores_ (top) and slopes of the linear regressions within the range [−60 ms, +60 ms] (bottom) computed for each gap factor. Grey lines show individual results and error bars indicate inter-individual SEM. Comparisons were done using paired t-tests.

A total of 10793 out of 11520 recorded trials were analyzed (93.7%). Applying the adaptive low-pass filter on latencies left 86 saccades in the wrong direction across participants, meaning that only 0.80% of anticipatory saccades in the correct direction remained in the latency distributions. Since the latencies for leftward and rightward responses did not differ–no main effect (*F*(1,11) = 0.05, *p* = 0.83) and no interaction with either Gap factor (*F*(1,11) = 0.33, *p* = 0.58) or SOA (*F*(4,44) = 0.05, *p* = 0.99)–we pooled them together in further analyses. Latency n_scores_ were finally computed for each of the nine [Gap factor × SOA] conditions taking the *No beep* condition as a baseline (the 0-ms SOA condition being the same for both Gap factors). The effects of auditory beeps on saccade-onset time as a function of SOA and Gap factor are plotted in the left panel of **Fig 3**. Gap factor (*F*(1,11) = 7.07, *p*<0.025, *η*_*p*_^*2*^ = 0.39) and SOA (*F*(4,44) = 268.27, *p*<0.0001, *η*_*p*_^*2*^ = 0.96) had a significant main effect on the median latency n_scores_. More importantly, the interaction was highly significant (*F*(4,44) = 97.17, *p*<0.0001, *η*_*p*_^*2*^ = 0.90), which indicates that introducing a gap/overlap modified the effect of the beep on SRTs differently depending on SOA.

To evaluate the extent of the warning/facilitatory effect when a beep was delivered near target onset, we performed single sample t-tests on the n_scores_ in the 0-ms SOA reference condition and found that saccade latencies decreased by about 17.1 ms when target and beep were presented together (*p*<0.0001). To characterize the beep-related modulation effect of SRTs for each of the Gap factors, we computed paired t-tests on the n_scores_ between the 0-ms SOA reference condition and the other SOA conditions. Saccade latencies were significantly shorter for beeps preceding target onset compared to synchronous beeps (*p*<0.0001 for all negative SOAs, except for SOA = −120 ms with *No gap*) and longer for beeps following target onset (*p*<0.0001 for all positive SOA). This replicates our previous findings [20], showing that a beep delivered simultaneously with the visual target shortened saccade latency (warning effect); and that a beep delivered before/after target onset reduced/increased saccade latency, compared to a simultaneous beep (modulation effect).

To compare the impact of the added overlap/gap on the beep-related modulation effect of SRTs between compensation factors, we performed three analyses. First, we computed paired t-tests to compare *No gap* with *Gap compensated* conditions for each of the SOA conditions (except SOA = 0 ms). We found a significant difference in all comparisons (p<0.0001 for all), indicating that the modulation effect was larger in the *Gap compensated* conditions at all SOAs. Second, we computed the modulation amplitude of latency n_scores_ over the entire SOA range and compared it between levels of the Gap factor (upper-right plot of **Fig 3**). We found that the modulation was significantly greater (*t*(11) = 14.90, p<0.0001) when the illusory gap was compensated (3.47) than when it was not (no gap; 1.33). Third, we computed individual linear regressions in the range [−60 ms, +60 ms] where variation is maximal (lower-right plot of **Fig 3**) and found again that the slope with the gap compensated (17.21) was significantly higher (*t*(11) = 7.37, p<0.0001) than without gap (8.96). Altogether, these results indicate that adding a delay between the onset of the target and the offset of the fixation point to compensate for the illusory gap/overlap produced by the beep-target multisensory temporal integration resulted in a huge increase of the saccade latency modulation by the beeps, just as expected. Thus, a beep delivered before target onset reduced SRTs even more when a gap was introduced between target onset and fixation offset, while a beep delivered after target onset increased SRTs even more with an additional overlap between target onset and fixation offset. These first findings support our initial hypothesis according to which an illusory gap/overlap induced by multisensory temporal integration reduces the impact of an irrelevant auditory stimulus on SRTs [20].

**Fig 4:**
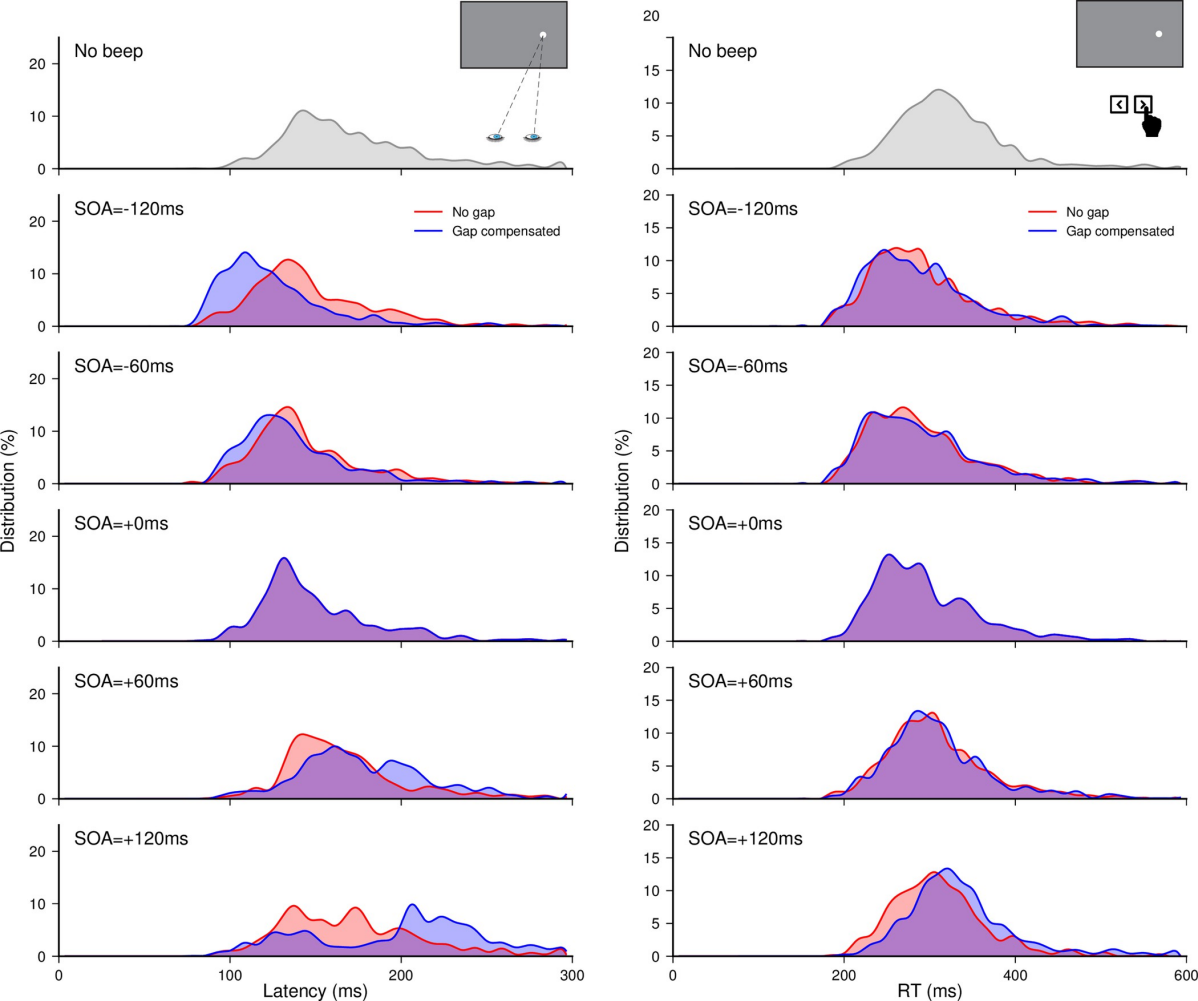
Experiment 1a and 1b. Saccade latency (left) and manual reaction time (right) distributions. Distributions for the *No beep* condition and for the *No gap* and *Gap compensated* conditions plotted for each SOA, with data pooled from all participants.

Finally, we looked for specific signatures of audiovisual interactions and gap/overlap effect in the distributions of saccade latencies. The distributions pooled together across all participants are presented in the left panel of **Fig 4**. When the beep followed target onset (positive SOAs), there was an increase in the spread of the distributions in the *No gap* and *Gap compensated* conditions, which reflected a strong reduction in the proportion of short- and regular-latency saccades and the emergence of a new population of longer-latency saccades–at the longest 120 ms SOA in the *Gap compensated* condition, this resulted into bimodal distributions. Conversely, when the beep was played before (or simultaneously with) the target, there was an increase in the proportion of short-latency saccades, and even more so at longer negative SOAs and in the *Gap compensated* conditions, although this did not induce an increase in the spread of the distributions due to floor effects. To confirm these observations with statistics, we performed the same analyses as for the medians on the median absolute deviations (MAD, see **S1 Fig**). There was no significant effect of a beep delivered simultaneously with target onset, as estimated by comparison with the *No beep* baseline, but there was a modulation effect over the tested SOA range, that was similar to that observed for median latencies. The spread in latency distributions increased with SOA, being significantly larger for SOA = 120 ms in *No gap* conditions (p<0.005), and for SOA = 60 and 120 ms in *Gap compensated* conditions (p<0.002 and p<0.0001, respectively) compared to the 0-ms reference condition. However, the spread did not change significantly for negative SOAs, either in *Gap compensated* or *No gap* conditions, confirming our intuition that a floor effect was preventing very early saccades to occur. Finally, the larger modulation effect of MADs observed for the *Gap compensated* conditions compared to the *No gap* conditions was not statistically significant after Bonferroni correction (p = 0.049, 0.042, 0.035 and 0.087 for SOA = −120 ms, −60 ms, +60 ms and +120 ms respectively) even though a qualitative change appeared in the distribution. Thus, adding a physical gap/overlap between fixation offset and target onset resulted in qualitative changes in the modulation of saccade-latency distributions, but without enough statistical power to become significant.

#### Effect on manual responses (Experiment 1b)

**Fig 5:**
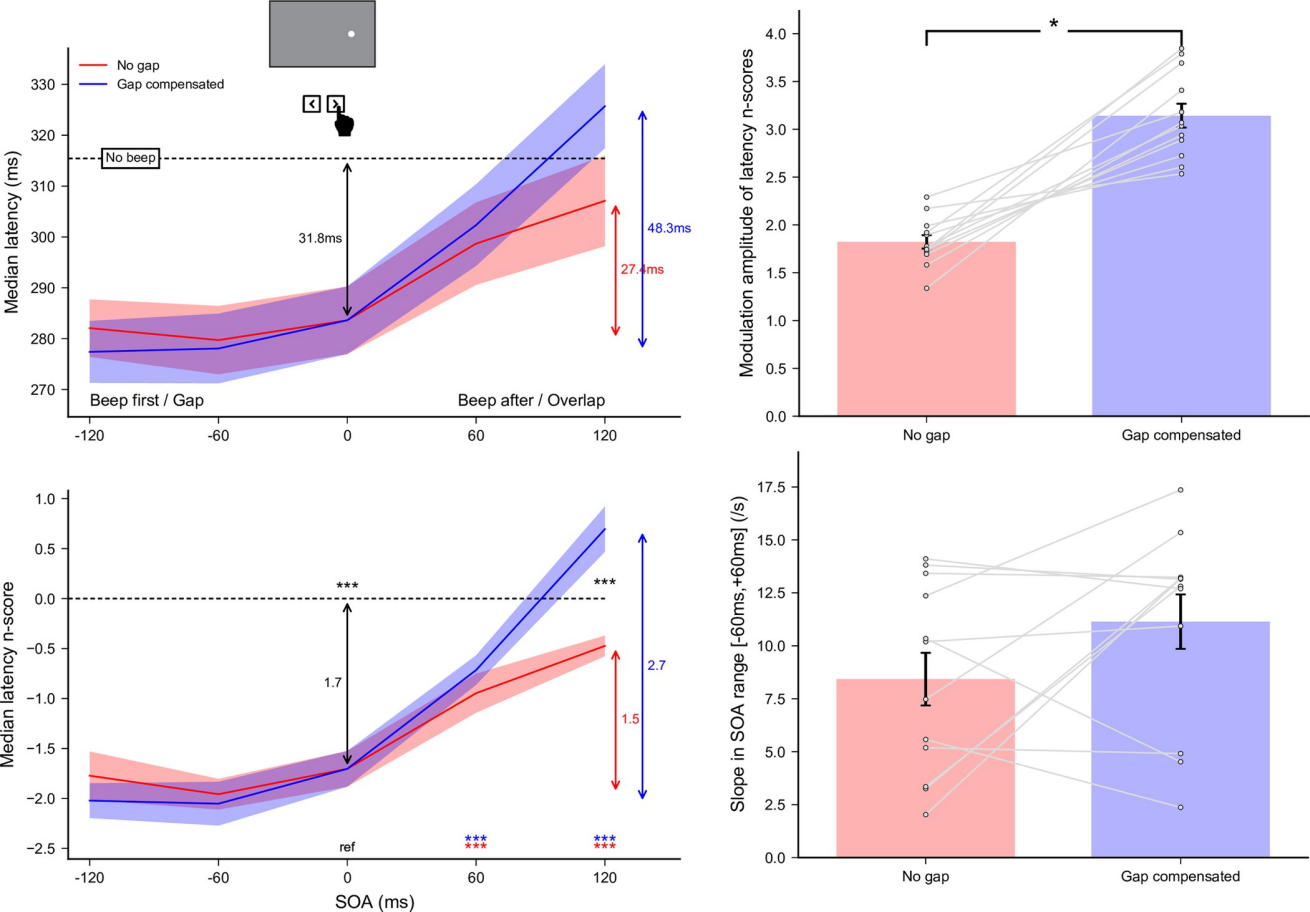
Experiment 1b. Effect of gap compensation on MRTs. **(Left panel)** Median latencies averaged across participants for the *No gap* and *Gap compensated* conditions as a function of SOA (top) and their corresponding n_scores_ (bottom). Dashed lines show the *No beep* baseline condition level. Statistics performed on the n_scores_ included a single sample t-test to compare the 0-ms SOA reference condition with the *No beep* condition (black arrow), and for each gap factor separately, paired t-tests compared this reference with each SOA condition (colored stars for each SOA above the X-axis). Finally, paired t-tests compared *No gap* and *Gap compensated* conditions for each SOA (black stars between curves). Three stars indicate highly significant differences for the paired t-tests after Bonferroni correction (p<0.00556). **(Right panel)** Modulation amplitude of latency median n_scores_ (top) and slopes of the linear regressions within the range [−60 ms, +60 ms] (bottom) computed for each gap factor. Grey lines show individual results and error bars indicate inter-individual SEM.

A total of 10633 out of 11520 recorded trials were analyzed (92.3%). Applying the adaptive low-pass filter on latencies left 110 responses in the wrong direction, which indicates that only 1.03% of anticipatory responses in the correct direction remained in the latency distributions. Since the latencies for leftward and rightward responses did not differ–no main effect (*F*(1,11) = 0.15, *p* = 0.71) and no interaction with either Gap factor (*F*(1,11) = 4.76, *p* = 0.052) or SOA (*F*(4,44) = 0.28, *p* = 0.89)–we pooled them together for further analyses. MRT n_scores_ were computed for each of the nine [Gap factor × SOA] conditions taking the *No beep* condition as a baseline (the 0-ms SOA condition being the same for both Gap factors). The effects of auditory beeps on manual responses as a function of SOA and Gap factor are plotted in the left panel of **Fig 5**. Gap factor (*F*(1,11) = 19.16, *p*<0.001, *η*_*p*_^*2*^ = 0.65) and SOA (*F*(4,44) = 73.36, *p*<0.0001, *η*_*p*_^*2*^ = 0.87) had a significant main effect on the median MRT n_scores_. More important, the interaction was significant (*F*(4,44) = 12.78, *p*<0.0001, *η*_*p*_^*2*^ = 0.54), suggesting that gaps affected MRTs differently depending on the SOA.

We found that MRTs decreased by about 31.8 ms when target and beep were presented together (*p*<0.0001) thus suggesting a substantially greater warning/facilitatory effect of the beep for manual responses in comparison with saccades, for either latency values or their n-scores (p<0.005 and p<0.0005, respectively, see **S2 Fig**). Regarding the beep modulation effect, we compared the n_scores_ between the 0-ms SOA reference and the other SOA conditions. MRTs were not shorter for beeps preceding target onset but they were longer for beeps following target onset (*p*<0.004 for all positive SOA), suggesting a modulation effect limited to conditions when beeps were delivered after the target. These results indicate that manual responses, just as saccades, were affected by an auditory beep, showing both a large warning effect, larger than for saccades, and a modulation effect occurring mostly for beeps delivered after target onset. A within-subject comparison of the modulation effect between manual responses and saccadic responses without physical gap yielded significantly greater n-scores for SRTs than MRTs (p<0.002), but no significant difference when contrasting latencies directly (see **S2 Fig**).

To compare the impact of the added physical overlap/gap on modulation effects of MRTs between compensation factors, we performed the same three analyses as for saccadic responses (**Fig 5**). First, we computed paired t-tests to compare latency n_scores_ between *No gap* and *Gap compensated* conditions separately for each SOA (except the 0-ms SOA) and found a significant difference only for SOA = +120 ms (p<0.0001). This finer analysis indicates that the significant interaction between Gap factor and SOA reported above stemmed almost exclusively from this SOA condition. Second, we computed the modulation amplitude of latency n_scores_ over the entire SOA range and compared it between Gap factors. We found that the modulation was significantly greater (*t*(11) = 7.77, p<0.0001) in the *Gap compensated* conditions (3.14) than in the *No gap* conditions (1.82). Once again, this difference resulted from the very large difference observed for SOA = +120 ms. Indeed, in a third analysis, we computed individual linear regressions in the range [−60 ms, +60 ms] and found that the slope for the *Gap compensated* conditions (11.14) was no different (p = 0.13) from that in the *No gap* conditions (8.43). Altogether, these results show that the addition of a physical overlap/gap to compensate for the illusory gap/overlap had a very limited effect on manual responses. Indeed, only when the beep was delivered 120 ms after target onset did the addition of a 60 ms overlap between target onset and fixation offset increase MRTs. Thus, the illusory gap/overlap induced by the multisensory temporal binding of the visual target and the beep only had a mild influence on manual responses.

Finally, distributions of MRTs pooled across all participants (right panel of **Fig 4**) revealed an increase in the proportion of early responses at null and negative SOAs, but this proportion did not vary between negative SOAs. Most importantly, MRT distributions, unlike SRT distributions, did not vary in shape between positive SOAs, and between *No gap* and *Gap compensated* conditions. Even the trend for bimodality observed for large fixation/target overlaps failed to emerge. Consistently, the median absolute deviation showed no significant beep-related effect, and no significant difference between *No gap* and *Gap compensated* conditions (see **S1 Fig**). Thus, adding a gap/overlap between fixation offset and target onset had virtually no impact on the shape of MRT distributions.

#### Not an anticipation effect

Delivering a beep synchronously with target onset (SOA = 0 ms) significantly reduced both SRTs and MRTs. It is noteworthy that participants sometimes triggered their response–whether ocular or manual–before they could process where the target would appear, and even more so when the beep was delivered before target onset. This could have reduced RTs at negative SOAs and produced the observed modulation. However, this possibility was ruled out by the adaptive low-pass filter applied to RTs (described in the Data processing section). Indeed, these anticipatory responses had equal chances of going in the right and in the wrong direction (50%). The latency cutoff was set such that for all participants, but one, only 1% of the responses in the opposite direction remained, therefore leaving a maximum of about 1% of anticipatory responses in the right direction. In one participant, 6% of the saccades went in the wrong direction throughout the latency range. We decided to keep that participant because the still limited proportion of directional errors occurred uniformly across the SOA range and could not impact conditions differentially. **S3 Fig** shows individual histograms with the number of wrong direction responses for each condition, plotted before and after the low-pass filter. Since the analysis of medians is robust to outliers, we conclude that anticipatory responses did not contribute to the observed SRT and MRT modulations.

### Discussion

In the first experiment, we investigated the possibility that audio-visual temporal binding has specific influences on oculomotor responses. To this end, we tested whether a physical gap/overlap between fixation offset and target onset would affect how strongly an irrelevant auditory stimulus modulates the latency of saccadic and manual responses over a range of SOAs. We hypothesized that an illusory gap/overlap–resulting from the perceived time shift of target onset towards the beep–minimizes the effect of beeps on SRTs, and hence that the introduction of a physical overlap/gap would at least partially compensate for this interfering effect. We first found, in agreement with this hypothesis, that adding a real overlap/gap did increase the beep-related modulation of SRTs across the entire range of tested SOAs (Experiment 1a). Moreover, this overlap/gap effect was essentially observed for saccades. Although the beep-related modulation effect increased significantly for both ocular and manual responses in gap compensated conditions, for the latter this was mostly due to the largest overlap condition. Indeed, the slope of the modulation observed in the [−60 ms, +60 ms] range of SOAs did not increase for manual responses, but it did drastically increase for ocular responses. Moreover, as indicated by the absence of qualitative changes in the distributions of MRTs, the effect of an irrelevant auditory beep on manual responses was barely impacted by the addition of a real overlap/gap between fixation offset and target onset. Still, in conditions without a physical gap/overlap, the strength of the beep-related modulation observed for manual responses–likely not under the influence of an illusory gap/overlap–was not much larger than for saccades; only was the beep warning effect (at null SOAs) substantially larger.

Based on the present findings, we can already draw the following conclusions. First, multisensory temporal binding modifies the perceived timing of visual events relative to one another (target onset relative to fixation offset) and results in illusory gap/overlap conditions that specifically affect oculomotor responses. The illusory gap/overlap would be responsible for the fact that an irrelevant auditory beep has a much smaller effect on the latency of visually guided saccades [15, 17, 19, 20] than what would be expected based on findings in a purely perceptual task [3]. This suggests that audiovisual interactions tend to be underestimated in saccade-targeting tasks due to temporal binding of visual and auditory events. Second, the illusory gap/overlap effect entertains similarities with the real/physical gap/overlap effect: it is much weaker for choice manual responses than saccades [28, 29, 42]. The gap/overlap effect is commonly assumed to involve two components, a first non-specific warning-type component, and another fixation-disengagement component, specific to the oculomotor system [e.g., 24]. Although the irrelevant auditory beep also likely acts as a warning event [17, 19, 20], the interference caused by the illusory gap/overlap essentially observed in saccades most likely reflects fixation-type mechanisms in the SC, and downstream of the SC, where saccades are programmed, thus explaining why this had little impact on manual responses which involve premotor and motor cortices. As audiovisual information combines in the intermediate layers of the SC, as well as FEF, for saccadic control, the possibility arises that multisensory integration and illusory gap/overlap effects interact, although our analysis of saccade-latency distributions suggested two independent effects. This question is addressed in Experiments 2 and 3. Experiment 2 was additionally aimed at replicating our findings for saccades, using a large range of gap values.

## Experiment 2: Parametric compensation of the illusory gap/overlap

In Experiments 1a-b, we used an arbitrarily fixed gap factor that was half the SOA. However, the illusory gap/overlap produced by beeps could be stronger or weaker, and the compensation effect overestimated and underestimated respectively. In Experiment 2, we thus tested more levels of the gap factor to investigate whether the introduction of a gap/overlap influences the effect of an irrelevant auditory stimulus on saccade latencies in a linear fashion and whether there is a saturation effect. Accordingly, for larger gap factors we expected to find a stronger modulation of saccade latencies, at least up to a certain level, whereas for smaller gap factors we expected a weaker modulation. Finally, for positive gap factors–opposite to compensation–we expected to cancel or reverse the modulation effect.

Six volunteers (four women and two men) participated in this experiment, all from the same pool of participants as for Experiments 1a and 1b. Participants were between 19 and 42 years old (average 25.3) and all were right-handed. The design and procedure were similar to the previous experiment. Trial conditions were defined as the combination of three factors: [4 Gap factors (+½, 0 (*No gap*), −½, −1) × 5 SOAs (−120, −60, 0, +60, +120 ms) + *No beep* baseline] × 2 Target Sides (left and right). Whereas negative gap factors compensated for the illusory gap/overlap, the positive gap factor increased it instead. As in Experiment 1a, the task was again to perform a quick saccade toward the target. Participants completed eight sessions of 216 trials each, totalizing 1728 trials (48 per Gap factor × SOA × Direction). The order of the conditions was randomized within blocks of 54 trials. Between each session participants had a few minutes break to rest. After each block of 54 trials, participants could rest for a few seconds before resuming the experiment.

### Results

**Fig 6:**
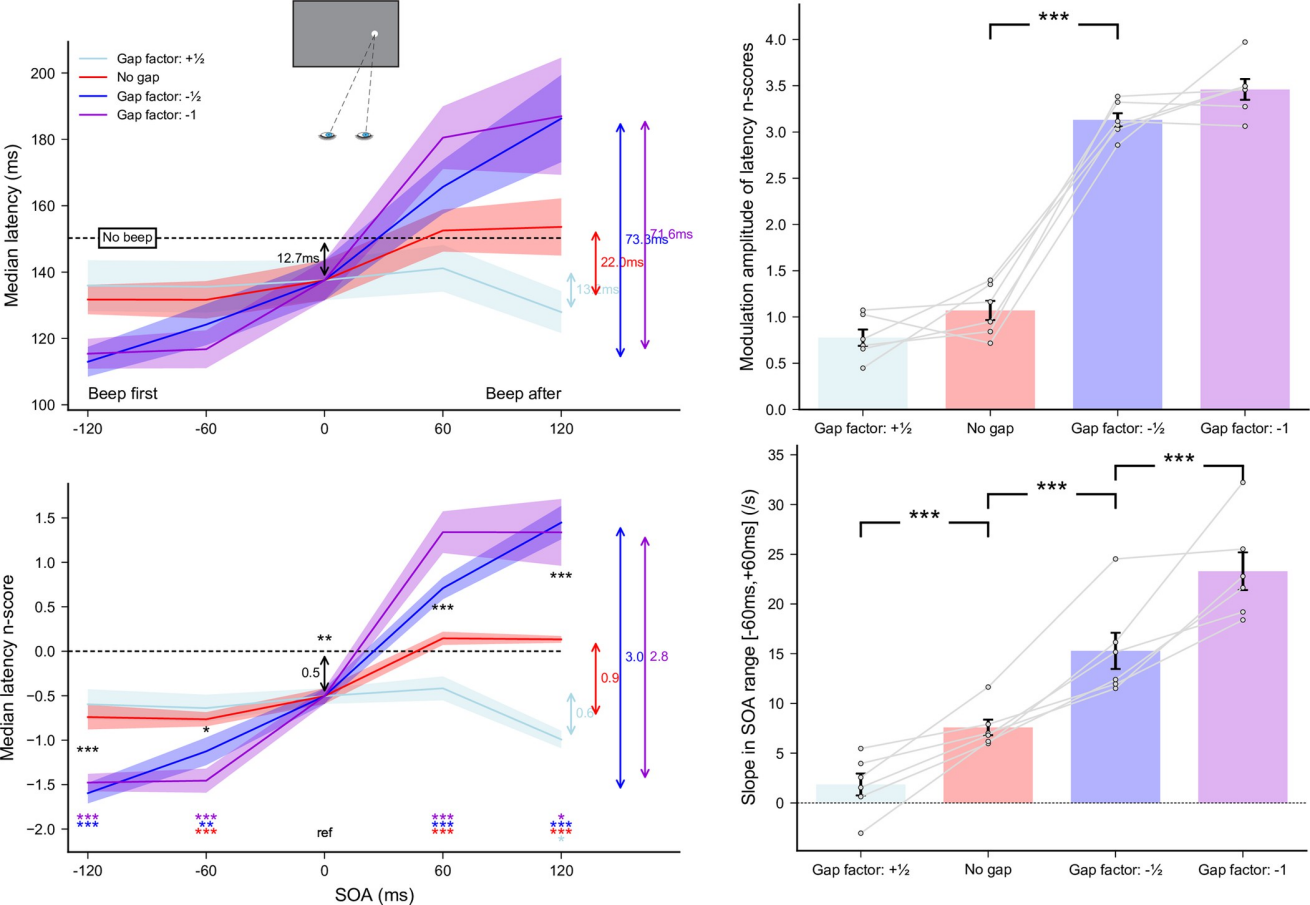
Experiment 2. Effect of various gap compensation factors on saccade latency. **(Left panel)** Median latencies averaged across participants for each gap factor in {+½, 0, -½, -1} as a function of SOA (top) and their corresponding n_scores_ (bottom). Dashed lines correspond to the *No beep* baseline condition. Statistics performed on the n_scores_ included a single sample t-test to compare the 0-ms SOA reference condition with the *No beep* condition (black arrow), and for each gap factor separately, paired t-tests compared this reference with each SOA condition (colored stars for each SOA above the X-axis). Three stars indicate highly significant differences after Bonferroni correction (p<0.00294) while single stars and two stars indicate significant differences with less (p<0.005) or without correction (p<0.05). **(Right panel)** Modulation amplitude of the latency median n_scores_ (top) and slopes of the linear regressions within the range [−60 ms, +60 ms] (bottom) computed for each gap factor. Grey lines show individual results and error bars indicate inter-individual SEM.

A total of 9974 out of 10368 recorded trials were analyzed (96.2%). Applying the adaptive low-pass filter on latencies left 18 saccades in the wrong direction, which indicates that only 0.18% of anticipatory saccades in the right direction remained in the latency distributions. Since the latencies for leftward and rightward responses did not differ–no main effect (*F*(1,5) = 0.03, *p* = 0.87) and no interaction with either Gap factor (*F*(3,15) = 1.92, *p* = 0.17) or SOA (*F*(4,20) = 0.75, *p* = 0.57)–we pooled them together for further analyses. Latency n_scores_ were finally computed for each of the 20 Gap factor × SOA conditions taking the *No beep* condition as a baseline, the 0-ms SOA condition being the same for all Gap factors. The effects of auditory beeps on SRTs as a function of SOA and Gap factors are plotted in the left panel of **Fig 6**. Gap factor (*F*(3,15) = 14.09, *p*<0.0002, *η*_*p*_^*2*^ = 0.74) and SOA (*F*(4,20) = 58.40, *p*<0.0001, *η*_*p*_^*2*^ = 0.92) had a significant main effect on the median latency n_scores_. More importantly, the interaction was also highly significant (*F*(12,60) = 35.02, *p*<0.0001, *η*_*p*_^*2*^ = 0.875), meaning that SOA affected SRTs very differently depending on the gap factor. In order to evaluate the extent of the facilitatory effect when a beep was delivered near target onset, we performed single sample t-tests with the n_scores_ observed in the 0-ms SOA condition, and consistently with Experiment 1a, we found that SRTs decreased by about 12.7 ms when target and beep were presented together (*p*<0.004).

To characterize beep-related modulation effects of SRTs separately for each of the Gap factors, we computed paired t-tests on the latency n_scores_ in order to compare the 0-ms SOA reference with the other SOA conditions. For null or negative Gap factors (0, −½ and −1), saccade latencies were significantly shorter for beeps preceding target onset compared to synchronous beeps (*p*<0.006 for all negative SOAs, except for SOA = −120 ms with *No gap*) and longer for beeps following target onset (*p*<0.003 for all positive SOAs, except for SOA = +120 ms with Gap factor = −1 where *p* = 0.01). For the positive gap factor (+½), there was no significant difference between the 0-ms SOA reference and the other conditions. This shows that beeps modulate saccade latencies, except when the added gap/overlap effect accentuates the illusory gap instead of compensating for it. To compare the beep-related modulation effects of SRTs between Gap factors, we performed three analyses (**Fig 6**). First, we compared the *No gap* and Gap factor = −½ for each of the SOA conditions (except the 0-ms SOA that is shared across Gap factors), and found a significant difference for SOAs of −120, +60 and +120 ms (p<0.003 for all) and a nearly significant difference for −60 ms SOAs (p = 0.023). In line with the findings of Experiment 1a, this analysis shows a larger beep modulation effect with a Gap Factor of ½ than with no gap. Second, we compared the modulation amplitude over the entire SOA range between the various gap compensation factors. While the modulation increased significantly from 0 to −½ Gap factors (1.07 vs. 3.13; *t*(5) = 15.70, p<0.00002), it was not different between +½ and 0 (0.78 vs.1.07; p>0.1) and between −½ and −1 (3.13 vs. 3.46; p>0.1). Thus, the modulation amplitude across the studied SOA range was not lower with anti-compensation than none, and it was not higher for strong compared to moderate compensation, reaching a maximum of 70 ms. The latter is explained by the SRT saturation for SOAs beyond +60 ms as well as below −60 ms. Third, we computed individual linear regressions in the [−60 ms, +60 ms] range and found slopes increasing significantly from +½ to 0 Gap Factors (1.9 vs. 7.6; *t*(5) = 4.85, p<0.005), from 0 to −½ Gap Factors (7.6 vs. 15.3; *t*(5) = 15.70, p<0.0001) and from −½ to −1 Gap Factors (15.3 vs. 23.3; *t*(5) = 3.65, p<0.015).

**Fig 7:**
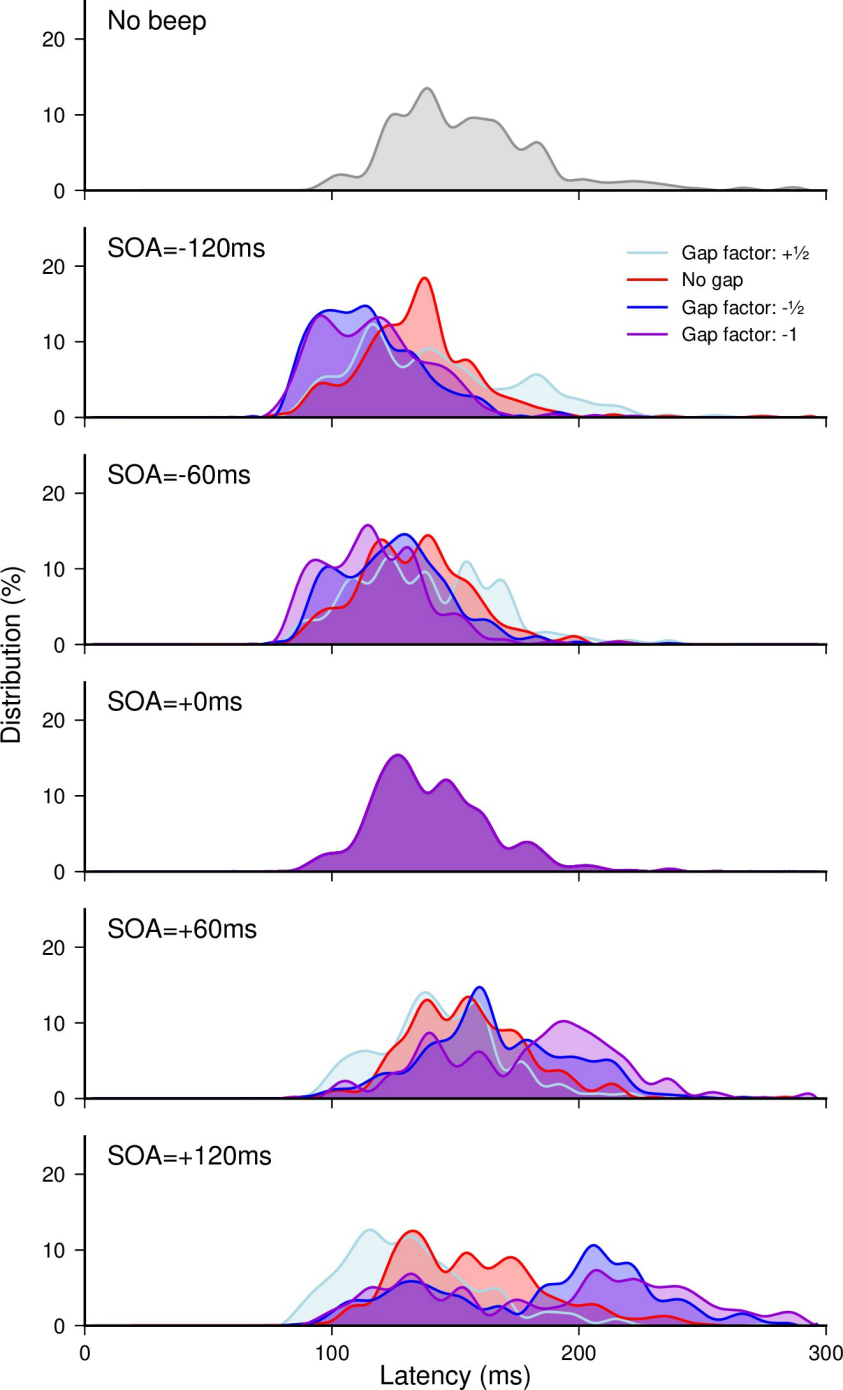
Experiment 2. Saccade latency distributions. Distributions for the *No beep* condition and for the different Gap factor conditions plotted for each SOA, with data pooled from all participants.

Finally, when looking at the shape of latency distributions (**Fig 7**), we could make two observations. First, whereas the proportion of short-latency saccades was inflated in conditions with a gap (negative SOAs for −½ and −1 Gap factors, and positive SOAs for +½ Gap factor), the proportion of long-latency saccades increased in conditions with an overlap (positive SOAs for −½ and −1 Gap factors, and negative SOAs for +½ Gap factor), resulting into more widely spread distributions in these conditions than in the corresponding No-gap conditions. Second, there was a clear tendency for distributions to become bimodal for target/fixation overlaps of 60 ms or above, visible for SOA = +120 ms in the −½ and −1 Gap factor conditions and for SOA ≥ +60 ms in the −1 Gap factor. Interestingly, the bimodality observed with an overlap of 60 ms between target onset and fixation offset with the −½ Gap factor (corresponding to SOA = +120 ms), does not seem to appear as clearly with the +½ Gap factor (corresponding to SOA = −120 ms), likely due to very early beeps shrinking the latency distributions, providing yet another indication that both effects combine in a rather independent manner.

### Discussion

In the present experiment, we manipulated the physical gap/overlap between fixation offset and target onset to either increase (+½ factor) or reduce the impact of the illusory overlap/gap (−½ and −1 factors). We observed that the beep-related modulation of saccade latencies progressively increased across conditions ranging from anti-compensation to strong compensation (factors from +½ to −1) for SOAs between −60 ms and +60 ms, but not beyond. Indeed, this modulation saturated with the strongest compensation for +/-120 ms SOAs, reaching a plateau. Altogether, these results indicate that the gap/overlap effect adds up to the audiovisual effect in a rather linear fashion, which points to an independence of the underlying mechanisms. Whereas negative gap factors did compensate for the illusory gap/overlap, the positive gap factor produced–as expected–the opposite effect, canceling almost entirely the beep-related modulation of saccade latencies across the entire SOA range. We could not estimate the illusory gap/overlap separately for each individual and condition. In Experiment 1a, we thus set the gap factor arbitrarily to –½ of the SOA, meaning that the compensation could have been over/underestimated. In the present experiment, we tested several gap factors to test whether our previous findings would also hold with other compensation factors. We found that the multisensory modulation of SRTs does increase or decrease in a linear fashion according to the gap factor. However, the extent of this modulation seemed to saturate, being no greater than about 70 ms, as with our initial –½ gap compensation factor. In the next experiment, we had a closer look at the linear additivity of beep-related modulation and gap/overlap effects and their corresponding independence, by measuring these effects separately and testing whether the linear combination of these two effects would yield an effect comparable to that obtained in Experiment 1a in which both factors were manipulated simultaneously.

## Experiment 3: Beep-related and gap/overlap effects are independent

So far, we always manipulated the gap (between target onset and fixation offset) together with the audiovisual SOA (between beep and target onset). As discussed earlier, the illusory gap/overlap went in the opposite direction to the expected beep-related multisensory effect. We designed this last experiment to compare directly–using the same methodology–the beep-related effect as a function of the SOA between the beep and the visual target, with the classical gap/overlap effect as a function of the delay between fixation offset and target onset (or gap duration). The goal was to look for similarities and differences between these two effects on saccadic responses. Furthermore, results from Experiment 2 suggested that beep-related and gap/overlap effects add up linearly–as illustrated with increasing gap factors–in a rather independent manner. This was further tested here, by comparing the two effects combined with the gap compensated condition of Experiment 1a on the same participants. If the mechanisms underlying audiovisual and gap/overlap effects on SRTs involve different mechanisms and/or different brain areas, this should translate into distinct signatures at a behavioral level, namely in the shape of the latency distributions and in a linear additivity of these effects.

Six volunteers (four women and two men) participated in this experiment, all from the same pool as in Experiments 1a and 1b. They were between 19 and 42 years old (average 27.0) and all were right-handed. Trial conditions were defined according to the following design: [5 Gap durations (+60, +30, 0, −30, −60 ms) + 5 SOAs (−120, −60, 0, +60, +120 ms)] × 2 Target Sides (left and right). In this experiment, the gap and audiovisual SOA factors were *not* manipulated jointly. For direct comparison with the results of Experiment 1a, the tested gap/overlap values were again set to minus half of the SOA values, even though in the *Gap conditions* no beep was ever delivered, and in the *Beep conditions* the onset of the target always coincided with fixation offset (no gap/overlap). The task was again to perform a quick saccade toward the target. Participants completed four sessions of 240 trials each totalizing 960 trials (48 per condition). The order of the conditions was randomized within blocks of 60 trials. Between each session participants had a few minutes break where they could rest. After each block of 60 trials, participants could rest for a few seconds before resuming the experiment.

### Results and discussion

**Fig 8:**
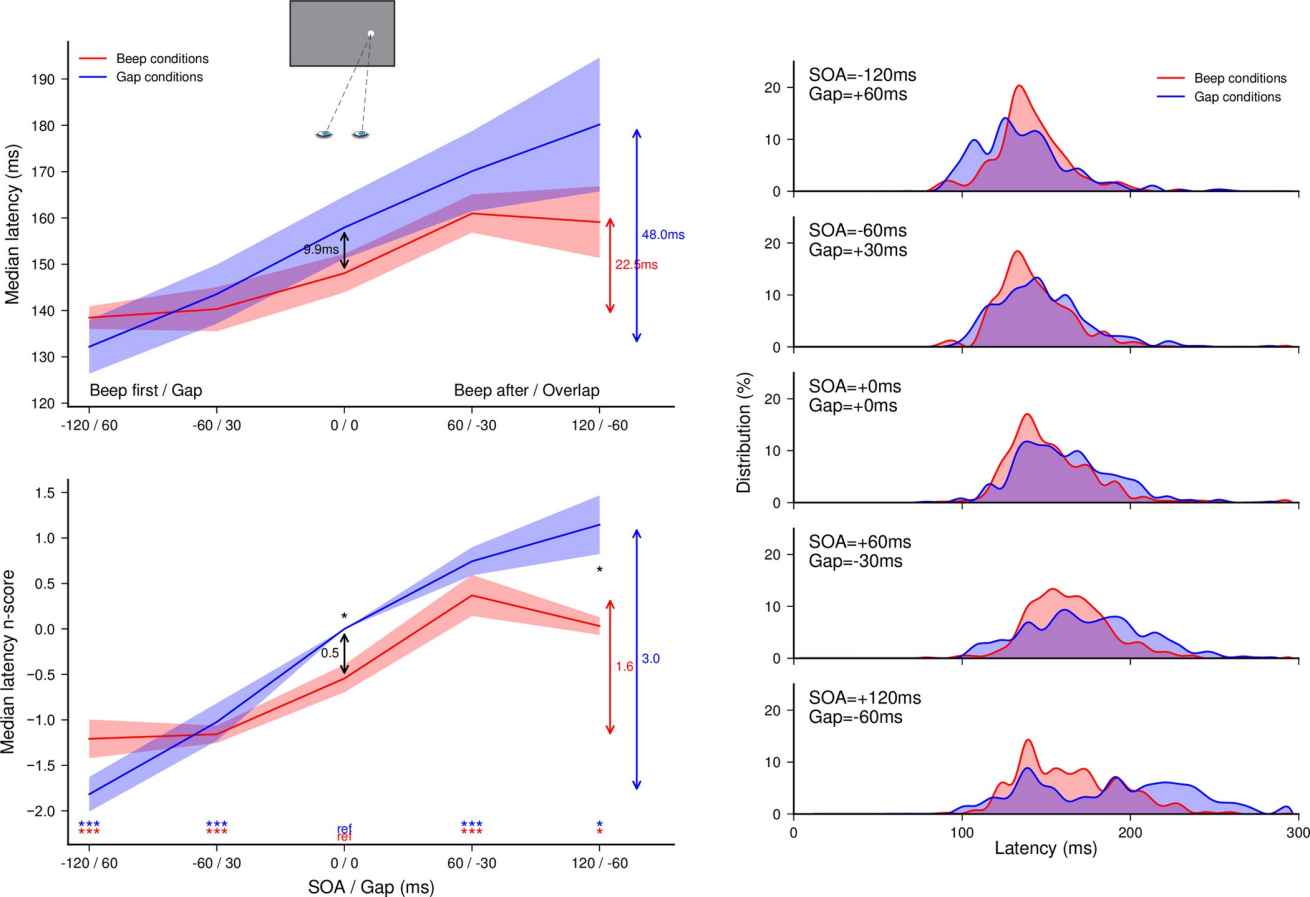
Experiment 3. Comparing the effects of beep MSI modulation and gap/overlap on saccade latency. (Left panel) Median latencies averaged across participants for the *Beep conditions* (no gap/overlap) and the *Gap conditions* (no beep) as a function of SOA or gap duration (top), and their corresponding n_scores_ (bottom). Statistics performed for each group of conditions separately on the n_scores_, included paired t-tests comparing each SOA or gap condition with the 0-ms SOA or Gap duration reference conditions, respectively (colored stars above the X-axis). Three stars indicate highly significant differences after Bonferroni correction (p<0.0125) while single stars indicate significant differences without correction (p<0.05). (Right panel) Saccade latency distributions for the *Beep conditions* and the *Gap conditions* plotted for each SOA and Gap, respectively, with data pooled from all participants.

A total of 5625 out of 5760 recorded trials were analyzed (97.7%). Applying the adaptive low-pass filter on latencies left 12 saccades in the wrong direction, which indicates that only 0.21% of anticipatory saccades in the correct direction remained in the latency distributions. Since the latencies for leftward and rightward responses did not differ–no main effect and no interaction with SOA/Gap in *Beep* (*F*(1,5) = 0.06, *p* = 0.81) and *Gap conditions* (*F*(1,5) = 0.03, *p* = 0.87)–we pooled them together for further analyses. Latency n_scores_ were computed for each of the 10 (Gap duration + SOA) conditions taking the 0-ms Gap condition as a baseline; the latter being the same as the previous *No beep* condition. For these experimental data, we analyzed separately *Beep* and *Gap conditions* before comparing the two. Both the effect of auditory beeps as a function of SOA (red curve) and the effect of gap/overlap as a function of gap duration (blue curve) are plotted in the left panel of **Fig 8**.

SOA had a significant main effect on the median latency n_scores_ (*F*(4,20) = 22.04, *p*<0.0001, *η*_*p*_^*2*^ = 0.88). SRTs were shorter for beeps preceding target onset (*p*<0.01 for all), and longer for beeps following target onset (*p*<0.002 for SOA = +60 ms and *p* = 0.033 for SOA = +120 ms), compared to synchronous beeps, thus confirming that beeps did modulate saccade latencies, in line with our above findings. The facilitatory effect associated with a beep delivered near target onset was of about 9.9 ms, although this was no longer significant after Bonferroni correction (*p* = 0.021). Gap duration (excluding the 0-ms Gap condition) also had a significant main effect on the median latency n_scores_ (*F*(3,15) = 78.35, *p*<0.0001, *η*_*p*_^*2*^ = 0.94). Saccade latencies were significantly shorter for gaps of 60 ms (*p*<0.0005) and 30 ms (*p*<0.006), and longer for overlaps of 30 ms (*p*<0.007) and 60 ms (*p* = 0.025), compared to the 0-ms gap condition. This shows that the gap/overlap duration also modulates saccade latencies, replicating with our setup the well-known effect first described half a century ago [23].

Saccade latency distributions in beep and gap conditions are represented for the different gap/SOA values, in the right panel of **Fig 8**. In the *Beep conditions*, there was only a slight increase in the spread of the distributions with increasing target-beep delay (for SOAs of +60 ms and above), replicating to some extent the results of Experiment 1a. However, there was an even greater increase in the spread of the distributions in the overlap conditions compared to the step condition. Furthermore, in the +60 ms overlap situation, a bimodal distribution tended to emerge, as in Experiment 1a, therefore suggesting that the above-reported trend for bimodal distributions at long target-beep SOAs in the *Gap compensated* conditions was essentially an effect of the added overlap between fixation offset and target onset rather than a beep-related effect. Statistical analysis of the latency median absolute deviation (see **S4 Fig**) indicated a strong increase in variability in the –30 and –60 ms *Gap conditions* compared to the no-gap condition (*p*<0.005 and *p* = 0.03), but no significant difference for the corresponding +60 and +120 ms *Beep conditions* compared to the no-beep condition (*p*>0.2). A direct comparison between *Gap* and *Beep conditions* confirmed that the spread was significantly greater for –30 and –60 ms *Gap conditions* than for the corresponding +60 and +120 ms *Beep conditions* (*p*<0.0025 and *p* = 0.046, respectively). Thus, we were able, simply based on the shape of saccade-latency distributions, to identify the specific signatures of audiovisual interactions and gap/overlap effects. Bimodality in the overlap conditions emerged as a result of a reduction in the proportion of short-latency saccades and an increase in the rightward tail of the distributions associated with longer-latency saccades. This is characteristic of conditions involving a foveal stimulus/event–here the delayed fixation offset–and likely reflects an inhibition of early-triggered saccades, and then recovery from this inhibition, which necessarily incurs additional delays for re- programming [53]. These different signatures provide clear evidence that beep and gap/overlap effects rely–at least partially–on distinct mechanisms.

**Fig 9:**
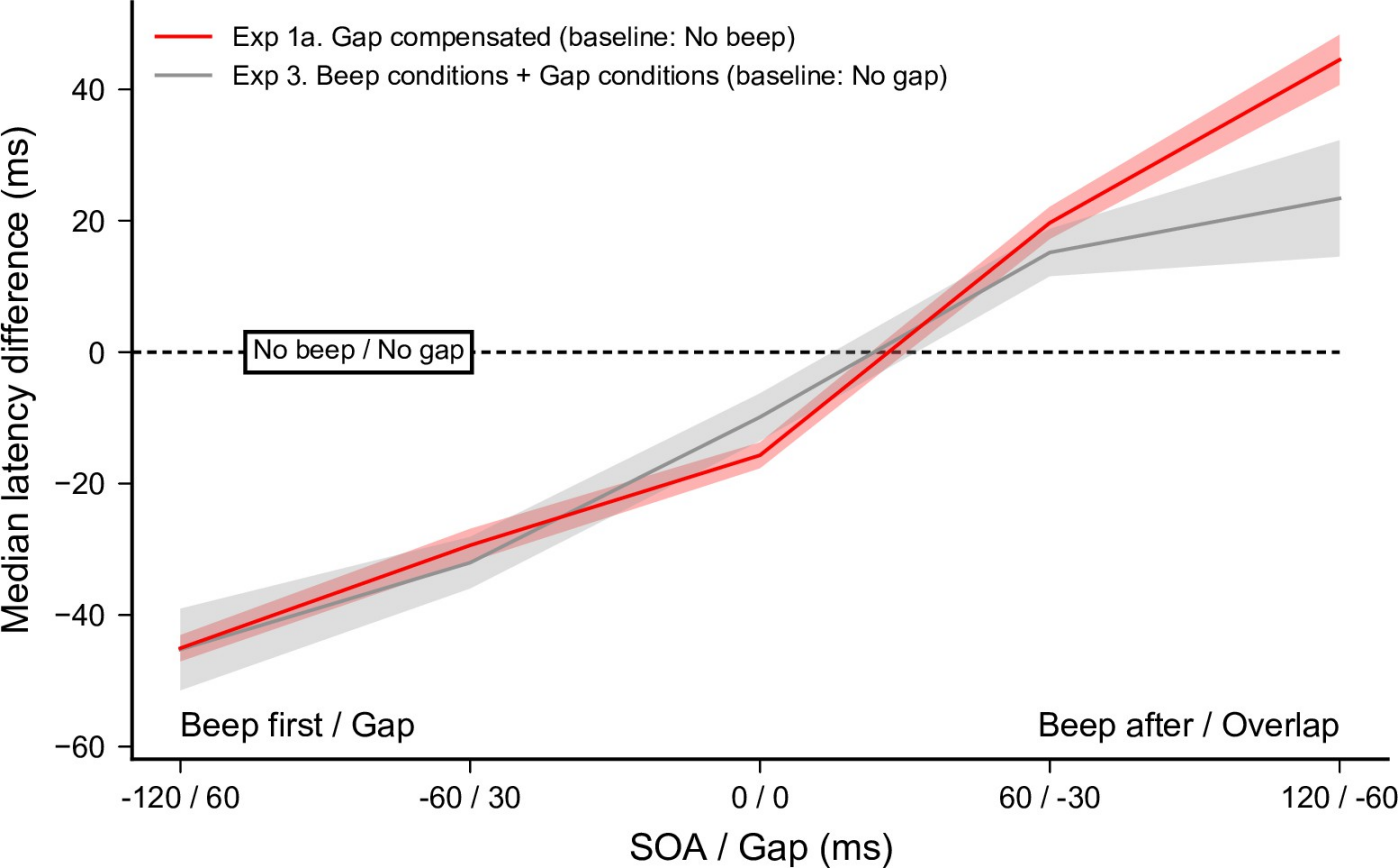
Experiment 3. Testing linear combination of beep MSI modulation and gap/overlap on saccade latency. Comparison of the *Gap compensated* condition from Experiment 1a for the same participants, with the combination of the *Beep conditions* and *Gap conditions* from Experiment 3, using the same baseline condition (respectively *No beep* and *No gap*).

In a last analysis, we checked whether the beep and gap/overlap modulation effects combine linearly. To this end, we compared the *Gap compensated* condition obtained for the same participants in Experiment 1a, with the linear sum of the effects obtained in the *Beep* and *Gap conditions* of the present experiment (see **Fig 9**), using the same baseline condition (*No beep* and *No gap*, respectively). None of the pairwise comparisons turned out significant (*p* = 0.98, 0.41, 0.064, 0.42 and 0.059, respectively) suggesting that the sum of both effects measured separately did roughly match the combined effect measured previously. Only for the largest overlap condition (60 ms) there seemed to be a potential difference, likely due to the strong variability in saccade latencies, as shown in the above distributions.

In this last experiment, we measured separately the audiovisual effect and the gap/overlap effect on saccade latency, using the same methodology and the same range of SOAs and gap durations as in Experiment 1a, and a subgroup of the participants. We found that both effects result in similar modulations of saccade latencies. However, adding the two effects together yielded the same results as the gap compensated condition in Experiment 1a in the same subset of participants, thus indicating that these effects add up almost linearly. We already had an indication of this linear combination in Experiment 2, in which we found that an increase in the gap factor leads to an increase in the total modulation. These findings, together with the above-reported differences in audiovisual and gap/overlap effects on the distributions of saccade latencies support the idea that multisensory and gap/overlap effects contribute to the control of saccadic eye movements in a largely independent manner. This has consequences on the underlying physiology: even though SC mechanisms may be involved in both phenomena, there seems to be very little interaction between the two.

## General discussion

How the brain combines inputs from different sensory modalities into a single percept, despite different afferent time course to multisensory areas, is a crucial issue in research on multisensory processing. Here we investigated whether and how audio-visual temporal binding affects motor responses, using two different effectors, the eye and hand. We showed that it has two competing, though independent/additive, effects on saccadic eye movements, but not manual key press responses: a direct multisensory effect, also observed for manual responses, and an indirect, deleterious, illusory-gap/overlap effect resulting from audiovisual temporal binding and affecting specifically saccadic responses. Implications for both multisensory and eye-movement research, as well as the underlying cerebral underpinnings are discussed. The possibility that there exist unconscious motor illusions, and not only conscious perceptual illusions, is also briefly raised.

### Audio-visual temporal binding induces an illusory gap/overlap that exclusively affects saccades

Several studies showed that irrelevant sounds can impact the latency of visually guided saccades, depending on when and where they are delivered. In general, a sound delivered near the onset time of a visual target, and at a spatially coincident location, tends to reduce saccade latencies, but more greatly for early beeps and less largely for late beeps [15–18]. This effect, also observed with unlocalized auditory stimuli [19, 20], is classically assumed to reflect a-specific warning-type processes [19] combined with multisensory integration mechanisms facilitating fixation/attentional disengagement [17]. We recently raised the possibility that audio-visual temporal binding may explain the modulation of SRTs with the beep-target delay [20]. However, since the modulation we estimated was far weaker than what we would expect based on perceptual temporal order judgments [3], we hypothesized that beeps shift the perceived timing of target onset, and that this results in two competing effects on saccade latencies (see **Fig 1**). The first is a multisensory modulation effect, that would, in line with the expected perceptual effect, induce a shortening/lengthening of latencies with early/late beeps. The second is an illusory overlap/gap effect, that results from target appearance being perceived earlier/later than fixation-point offset with early/late beeps, and that would induce an increase/decrease of saccade latencies, as consistently reported in oculomotor research [23]. The present findings are consistent with this assumption. In the first experiment, we added a real overlap/gap (50% of the SOA) to the classical audiovisual paradigm to compensate–at least partially–for the illusory gap/overlap. We found that this manipulation increased the beep-related modulation of saccade latencies across the entire range of SOAs (Experiment 1a). In a similar manner, we showed in Experiment 2 that we could increase, but also decrease, the modulation effect to different extents using different gap-SOA ratios. Our results therefore indicate, in line with our original assumption, that brief auditory stimuli induce an illusory gap/overlap, which attenuates the effect the same auditory stimuli should have on saccades. They provide clear evidence for a role of multisensory temporal binding in the previously reported relationship between the latency of visually guided saccades and the asynchrony between auditory and visual stimuli. Thus, a beep delivered near the time of a visual target may act as a warning event, but this warning effect is modulated by the perceived temporal sequence of visual and auditory events.

Importantly, the gap/overlap effect we observed for saccades did not generalize to choice-manual responses. In Experiment 1b, we found a modulation of MRTs with the delay between a visual target and an irrelevant auditory beep, but this remained unaffected by the addition of a physical gap/overlap between fixation offset and target onset. This finding is consistent with the literature reporting only mixed evidence for a gap/overlap effect in manual responses [19, 27, but see 28]. It suggests that the illusory gap/overlap effect, just like the physical gap/overlap effect, is for a great part specific to oculomotor responses and largely attributable to SC mechanisms involved in saccade programming. Visual offsets, when displayed together with or before a visual target, are visual cues that allow to be temporally prepared for a response, regardless of the effector. However, visual offsets, and in particular foveal offsets, also have specific, non-warning, effects on orienting responses [19, 27, 29, see also 30]. By reducing the activity of rostrally located SC neurons, which massively project onto downstream omnipause neurons responsible for fixation behavior [37], and reciprocally rising saccade-related activity [38], they greatly facilitate fixation disengagement, and hence saccade initiation [24, see also 22]. This SC component of the gap effect does specifically affect orienting responses [45, 46], but not manual keypress responses, which mostly involve primary and supplementary motor areas. The SC is a major multisensory structure, that integrates afferents from many cortical and subcortical brain regions, but all cortical inputs are from a single sensory modality [12]. It is therefore quite likely that the binding of visual and auditory signals, that induce an illusory gap/overlap effect in audio-visual saccade-target paradigms, originates in the SC.

Multisensory cells are found in the deeper layers of the SC. These neurons see their response enhanced when inputs from different sensory modalities are spatially coincident and fall within the neurons’ receptive field [54], pending the stimuli are presented within a reasonable time window [of about 100-200ms in anesthetized cats, 10, and 500ms in awake monkeys, 55]. Here we used an unlocalized beep as an auditory stimulus. The same acoustic signal was delivered binaurally and was likely interpreted as originating from straight-ahead. This stimulus therefore did not convey any information about where the target would appear. Whether and how this affected SC activity remains undetermined. However, studies have shown that the receptive fields of auditory responsive neurons in the SC are large, and larger than for visually responsive neurons [56], a finding which is consistent with the poorer accuracy in the spatial localization of sounds compared to visual signals reported in psychophysical studies [57]. Therefore, the receptive fields of visually responsive neurons coding for an 8-degree eccentricity (the eccentricity of visual targets in our experiments) may well overlap with the receptive fields of auditory neurons coding for central beeps and result in enhanced activity of corresponding audiovisual SC neurons. We may thus speculate, based on the present findings, that an early (late) beep enhanced neuronal activity in the SC map, and that this enabled earlier (delayed/greater) activation of neurons coding for the target location. The resulting overlap (gap) between saccade-related activity (associated with the target) and fixation-related activity (associated with the fixation stimulus) then made the balance shift most preferably towards fixation (move) behavior, leading in turn to an increase (decrease) in saccade latency. Investigations of neuronal activity in the SC using unlocalized sounds should help clarifying this issue.

### Auditory and visual events modulate SRTs independently: Similar neural substrates but different mechanisms?

The planning of goal-directed saccades can be influenced by auditory events (short beep) as well as visual events (fixation offset), through audiovisual interactions and gap/overlap effects respectively. At first sight, these influences seem very similar in that early sensory events, whether visual or auditory, shorten saccade latencies, whereas late events increase them. Both foveal offsets and auditory stimuli likely contribute to the disengagement of fixation in and downstream of the SC. However, our findings provide several pieces of evidence suggesting a clear independence between the mechanisms driving these two effects. In Experiment 2, we tested several levels of gap factors to test whether greater compensation of the illusory gap/overlap would produce an overall linear increase in the modulation of saccade latencies over the tested range of SOAs. We did find an increase in the overall modulation with increasing compensation (i.e., from anti to none, from none to mild and from mild to strong), but up to a certain level only. Indeed, for the strongest compensation–with a gap opposite to the SOA value–the modulation saturated beyond ±60 ms. This indicates that the gap/overlap effect adds up to the beep-related modulation effect rather independently. In Experiment 3, we decoupled these effects and measured separately both the beep-related modulation effect for a given range of SOAs (without gap/overlap) and the gap/overlap effect as a function of gap duration (without beep). Both effects modulated saccade-latency medians in similar ways. The sum of the latency medians for corresponding conditions in both sets resulted in a modulation that roughly matched that observed with the same participants in the gap compensated condition of Experiment 1a, suggesting that these effects are additive. We additionally observed, in all three experiments, that the (added) gap/overlap between fixation offset and target onset affected the distributions of saccade latency in a manner that did not compare with the beeps. Gaps, and even more so larger gaps, strongly inflated the proportion of very-short latency saccades (of about 100 ms), while (larger) overlaps led to a suppression of early saccades and a reciprocal increase in the proportion of long-latency saccades, leading to often bimodal distributions. Beeps more moderately affected the shape of the latency distributions, and never resulted in very-short (or extremely long) saccade latencies (and hence bimodal distributions), intervening much later during an eye fixation than the gap/overlap effect. The distinct behavioral signatures of beep and gap/overlap effects strongly suggest that they involve different neural mechanisms and/or that they intervene with different time courses.

Thus, audiovisual interactions and gap/overlap effects are similar in many ways. However, we gathered evidence suggesting that these effects rely on independent mechanisms, and that they combine linearly and can saturate. This has consequences on the underlying neurophysiology: even though the superior colliculus may be involved in both mechanisms, there seems to be very little interaction between the two. We above made the proposal that audiovisual temporal binding, responsible for the illusory gap/overlap effect, originates in the SC. The earliness of the (compensated) gap/overlap effect, as indicated by saccade latency distributions, corroborates this view. It additionally suggests that multisensory interactions and (resulting) visual-offset effects may reflect bottom-up processes that intervene early on to shape the SC activity pattern. However, this does not preclude audiovisual interactions in other cortical brain areas, like FEF, that are associated with visual selection and attention allocation, as well as fixation disengagement [58]. These higher-level and inevitably slower processes would intervene secondarily to modulate, through top-down projections to the SC, the onset time of saccadic responses according to the laws of multisensory temporal binding [3].

In our previous study [20], fixation offset and target onset were always concomitant (step paradigm), but some participants reported seeing gaps or overlaps depending on the timing of the beep relative to visual target onset. Based on these reports, we naturally called this effect illusory gap/overlap. However, since the term illusion usually refers to an effect acting at a conscious perceptual level, speaking of an illusory gap/overlap effect now turns out to be inappropriate. As we noted above, it is firstly an oculomotor effect that occurs at a very early processing stage, likely in the SC. At this stage, it is therefore likely only pre-conscious. The perceptual illusion reported by the participants would rather be a bottom-up effect that builds up later in frontal areas, such as FEF. Would the effect on saccade generation result from a top-down influence produced by this illusion, it would likely come with much longer delays, which was clearly not the case. Curiously, such a perceptual illusion did not influence manual responses despite these being generated after longer delays compared to saccadic responses. This may be explained by the dissociation between visual processing streams for perception and action, as illustrated by the famous Ebbinghaus illusion [59].

## Conclusions

To sum up, we found evidence supporting that only the warning component of the gap effect influences both manual and oculomotor behaviors, while the remaining component related to activity in the SC has no influence on choice manual responses. Moreover, the multisensory binding of auditory and visual-target related signals shows a similar modulation pattern for both manual and oculomotor behaviors, but it does produce an illusory gap/overlap that reduces the modulation only in the specific case of saccades. Eye-movements, because of their underlying multisensory neural substrates, as well as their shorter programming times, enable the study of multisensory processes with greater accuracy and less noise than any other motor effector. Researchers should yet be aware that audiovisual temporal integration induces an illusory gap/overlap that has deleterious effects on the speed of saccadic responses, at least in paradigms using transient auditory and visual stimulations: this reduces the multisensory effects in comparison with perceptual temporal judgment tasks. Based on our findings, we thus recommend avoiding saccades and using instead non-goal directed choice manual responses to avoid such interfering effects, unless the illusory gap/overlap effect in saccadic eye movements is precisely used as a way to investigate the mechanisms contributing to multisensory temporal binding in subcortical brain structures. In conclusion, the buzzing sound of a flying mosquito landing on our leg will likely increase the speed with which we initiate a saccade to foveate the insect, but it will less largely speed-up the choice of the most appropriate hand response used to crush the insect.

## Supporting information

S1 Table Modulation effect statistics. Pair-wised comparisons between the mean latency median n_score_ of each SOA condition with the reference condition (SOA = 0 ms, grey shaded columns). Statistical analyses followed 3 steps: a normality test (Shapiro-Wilk), a variance test (Levene) and a paired Student’s t-test (single value for the *No beep* condition). This table reports the outcome of all these tests together with the effect size (Cohen’s d).
(XLSX)

S2 Table Factor effect statistics. Pair-wised comparisons for each SOA condition between the mean latency median n_score_ of each gap factor and the zero gap factor (grey shaded columns). Statistical analyses followed 3 steps: a normality test (Shapiro-Wilk), a variance test (Levene) and a paired Student’s t-test. This table reports the outcome of all these tests together with the effect size (Cohen’s d).
(XLSX)

S1 Fig Experiment 1a (left) and 1b (right). Effect of sound beeps on saccadic and manual RT. Median absolute deviation of the response latency distribution averaged across participants for each SOA condition (top) and their corresponding n_scores_ (bottom). Dashed lines show the *No beep* baseline condition level. Statistics performed on the n_scores_ included a single sample t-test to compare the 0-ms SOA reference condition with the *No beep* condition (black arrow), and for each gap factor separately, paired t-tests compared this reference with each SOA condition (colored stars for each SOA above the X-axis). Finally, paired t-tests compared *No gap* and *Gap compensated* conditions for each SOA (black stars between curves). Three stars indicate highly significant differences for the paired t-tests after Bonferroni correction (p<0.00556).
(PDF)

S2 Fig Comparing warning effect (left column) and modulation effects (middle and right columns) between saccadic (Exp 1a) and manual RT (Exp 1b). Effect on the response latency median (top) and their corresponding n_scores_ (bottom) for individuals participants (gray lines) and averaged (bars with inter-individual SEM). Comparisons were done within-subject using paired t-tests, with an alpha value set to 0.5.
(PDF)

S3 Fig Responses going in the wrong direction of Experiments 1a (left panel) and 1b (right panel). Individual histograms plotting the number of initial responses opposite to where the target appeared for each SOA and *No beep* conditions. The **upper plots** shows the initial data with all the correctly detected responses. The **lower plots** shows the analyzed data after exclusion of anticipative responses by applying the optimal low-pass cutoff filter determined individually (see the Data processing section for further details).
(PDF)

S4 Fig Experiment 3. Effect of sound beeps on saccadic RT. Median absolute deviation of the response latency distribution averaged across participants for each SOA condition (top) and their corresponding n_scores_ (bottom). Statistics performed for each group of conditions separately on the n_scores_, included paired t-tests comparing each SOA or gap condition with the 0-ms SOA or Gap duration reference conditions, respectively (colored stars above the X-axis). Three stars indicate highly significant differences after Bonferroni correction (p<0.0125) while single stars indicate significant differences without correction (p<0.05). Finally, paired t-tests compared *Beep* and *Gap conditions* for each SOA (black stars between curves). Three stars indicate highly significant differences for the paired t-tests after Bonferroni correction (p<0.00556).
(PDF)
