# Supplementary figures and images for: Multisensory temporal binding induces an illusory gap/overlap that reduces the expected audiovisual interactions on saccades but not manual responses
Source: PLoS One. 2022 Apr 7;17(4):e0266468. doi: 10.1371/journal.pone.0266468 (PMC8989229; doi:10.1371/journal.pone.0266468)

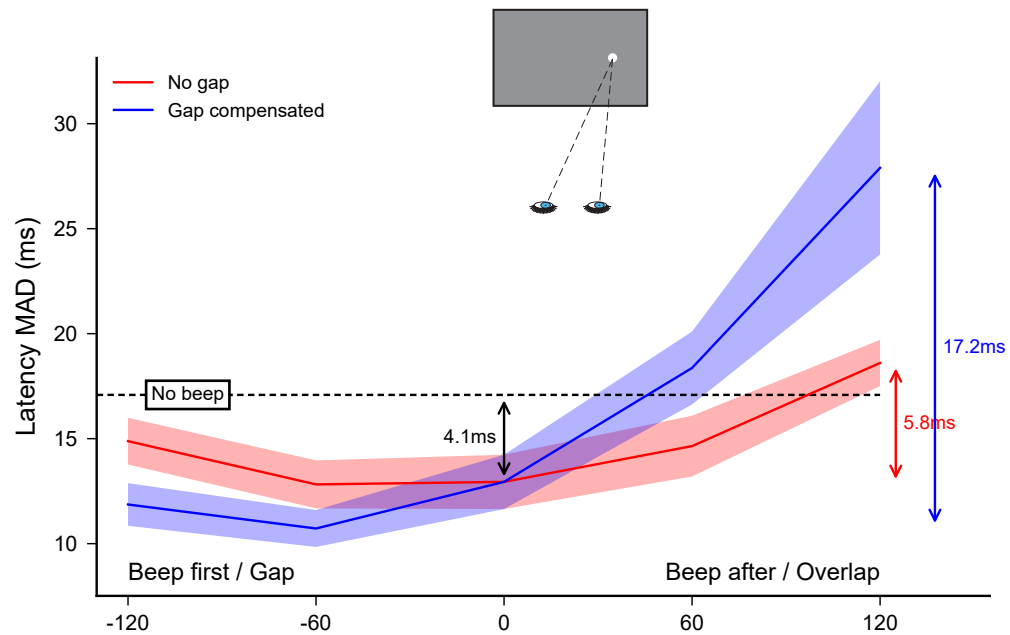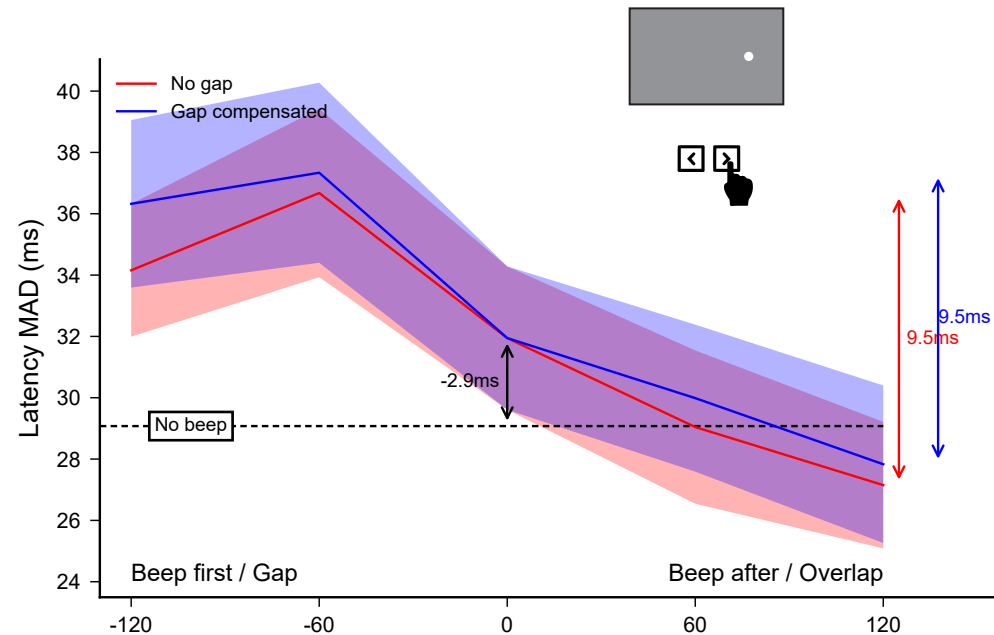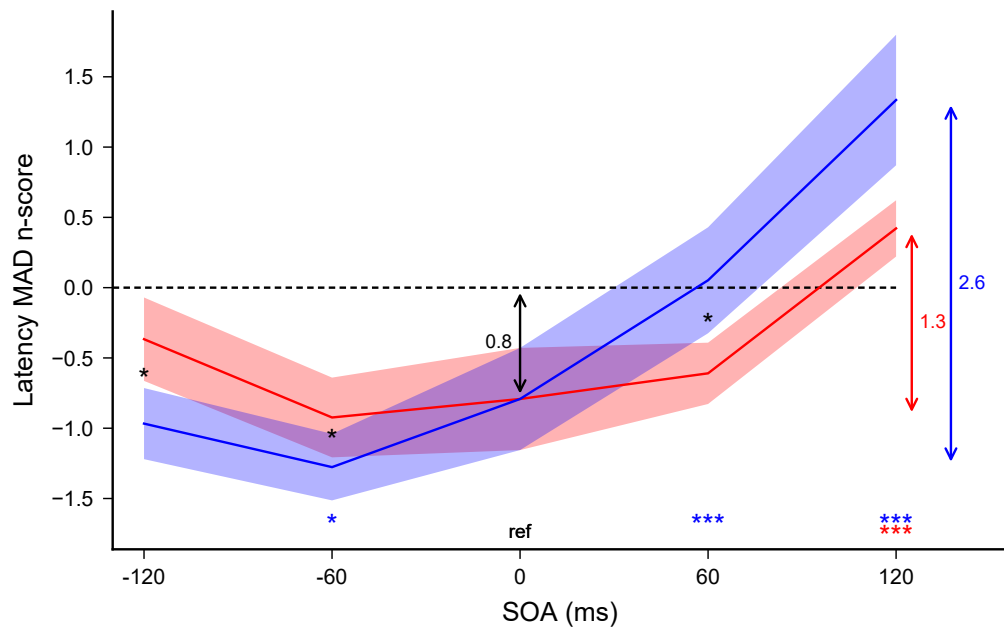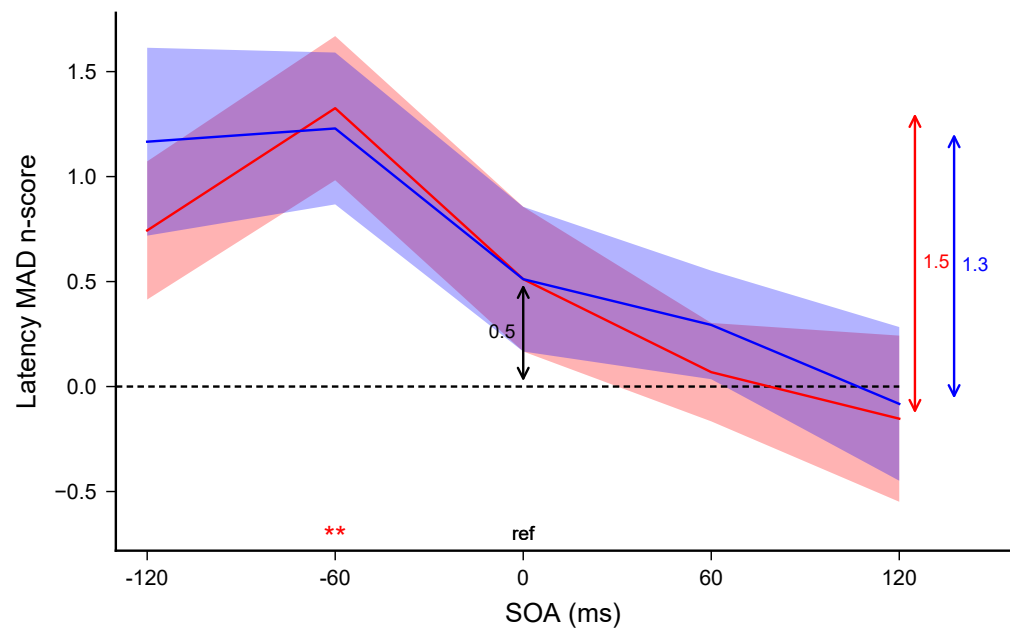

Supplement: S1 Fig — Experiment 1a (left) and 1b (right). Effect of sound beeps on saccadic and manual RT. Median absolute deviation of the response latency distribution averaged across participants for each SOA condition (top) and their corresponding nscores (bottom). Dashed lines show the No beep baseline condition level. Statistics performed on the nscores included a single sample t-test to compare the 0-ms SOA reference condition with the No beep condition (black arrow), and for each gap factor separately, paired t-tests compared this reference with each SOA condition (colored stars for each SOA above the X-axis). Finally, paired t-tests compared No gap and Gap compensated conditions for each SOA (black stars between curves). Three stars indicate highly significant differences for the paired t-tests after Bonferroni correction (p<0.00556). (PDF) [file pone.0266468.s003.pdf]

Signal effect

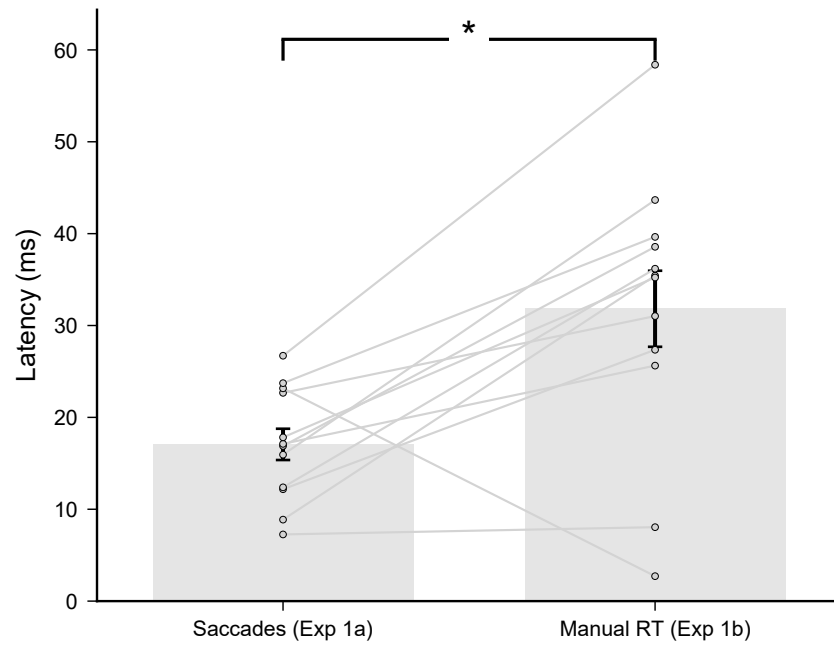

Modulation effect (No gap)

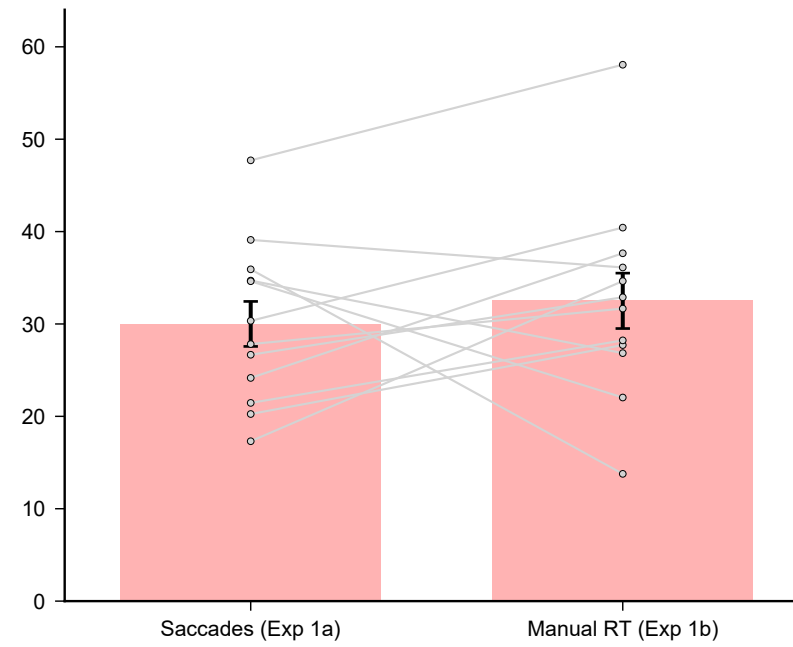

Modulation effect (Gap compensated)

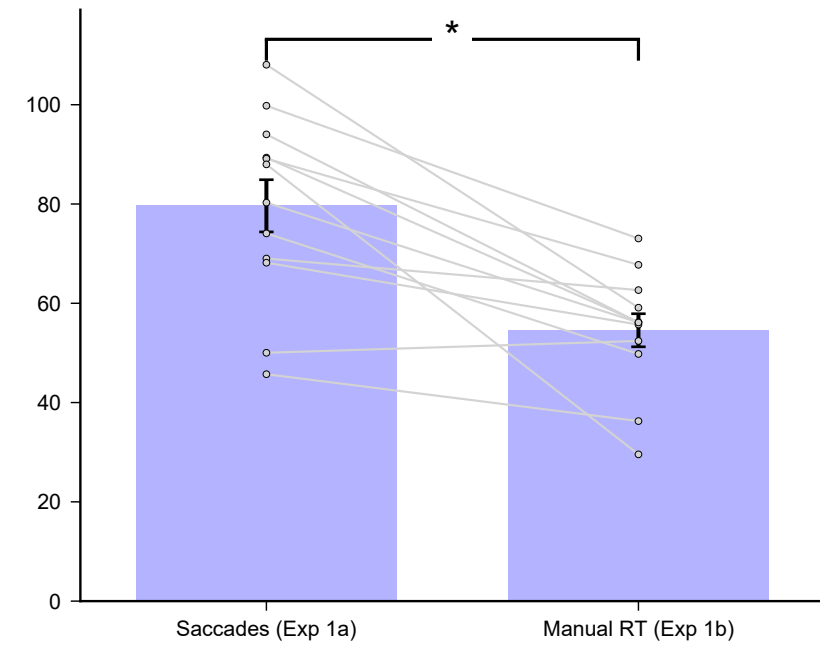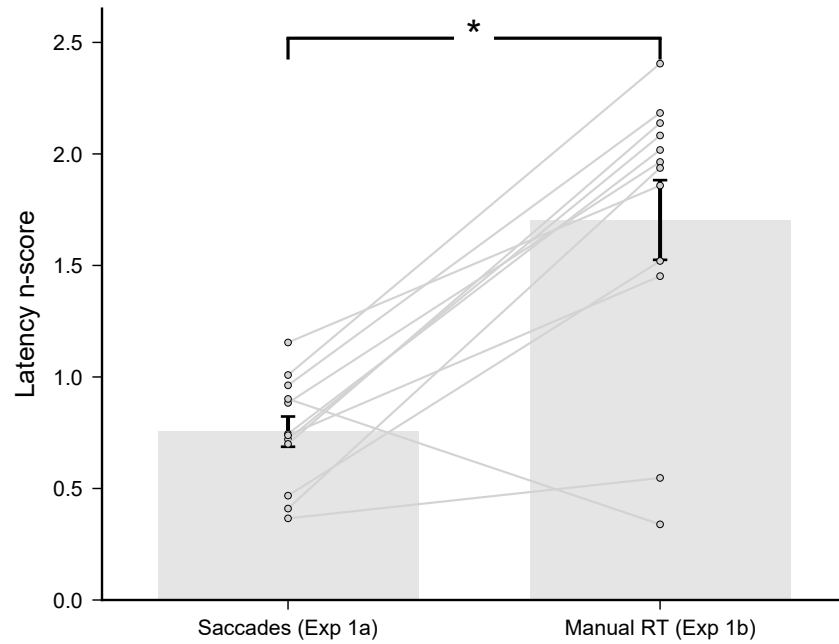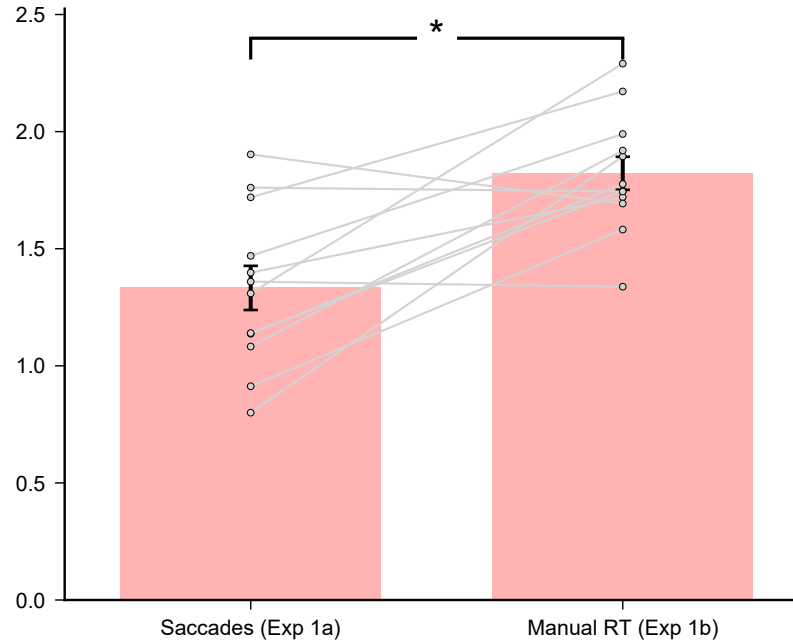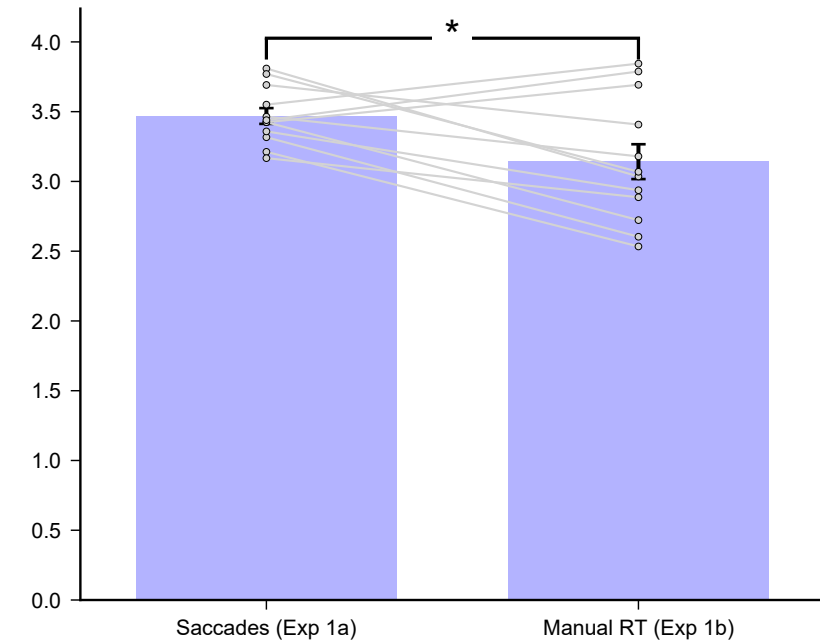

Supplement: S2 Fig — Comparing warning effect (left column) and modulation effects (middle and right columns) between saccadic (Exp 1a) and manual RT (Exp 1b). Effect on the response latency median (top) and their corresponding nscores (bottom) for individuals participants (gray lines) and averaged (bars with inter-individual SEM). Comparisons were done within-subject using paired t-tests, with an alpha value set to 0.5. (PDF) [file pone.0266468.s004.pdf]

Experiment 1a. Saccades

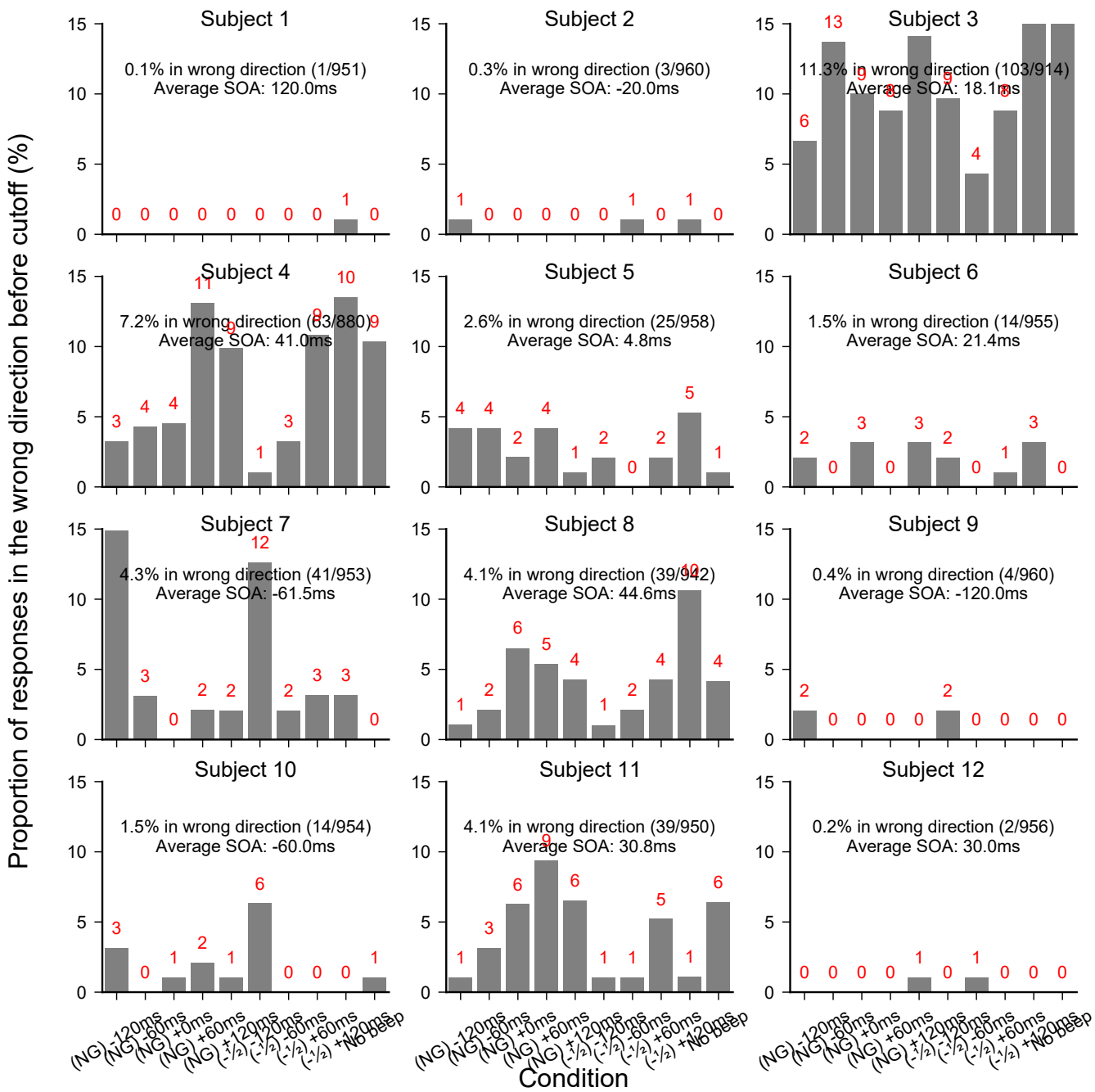

Experiment 2a. Manual responses

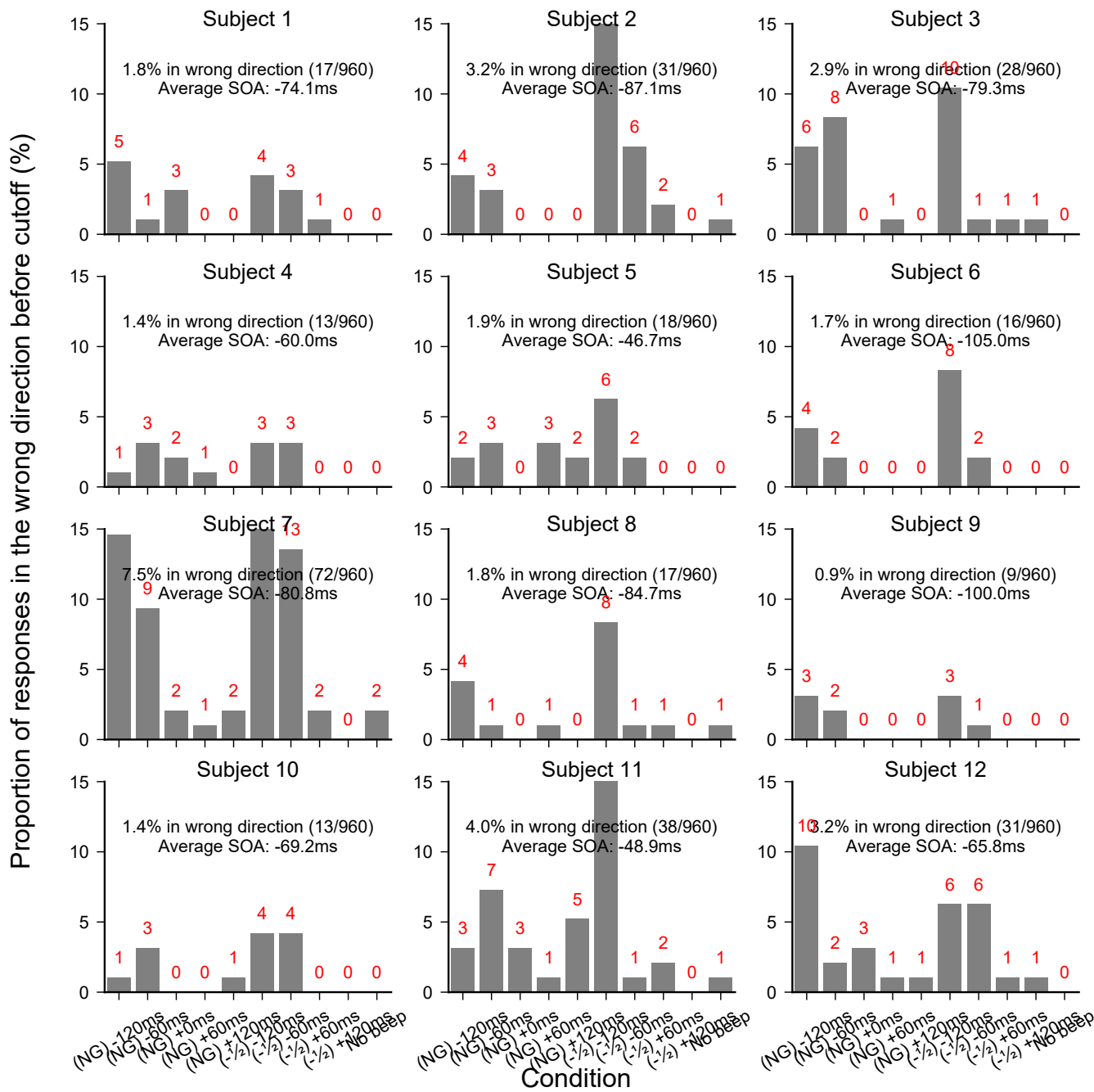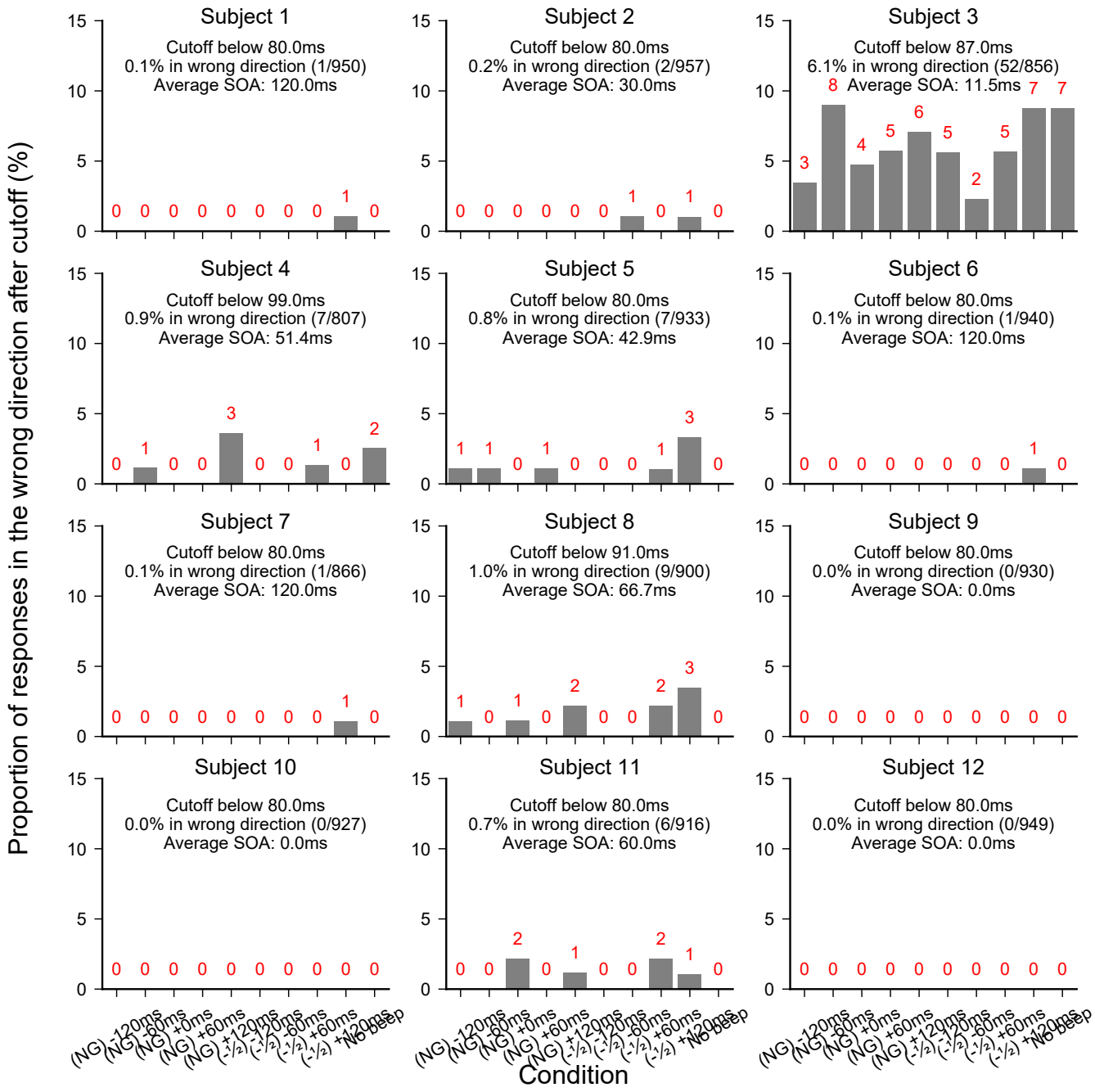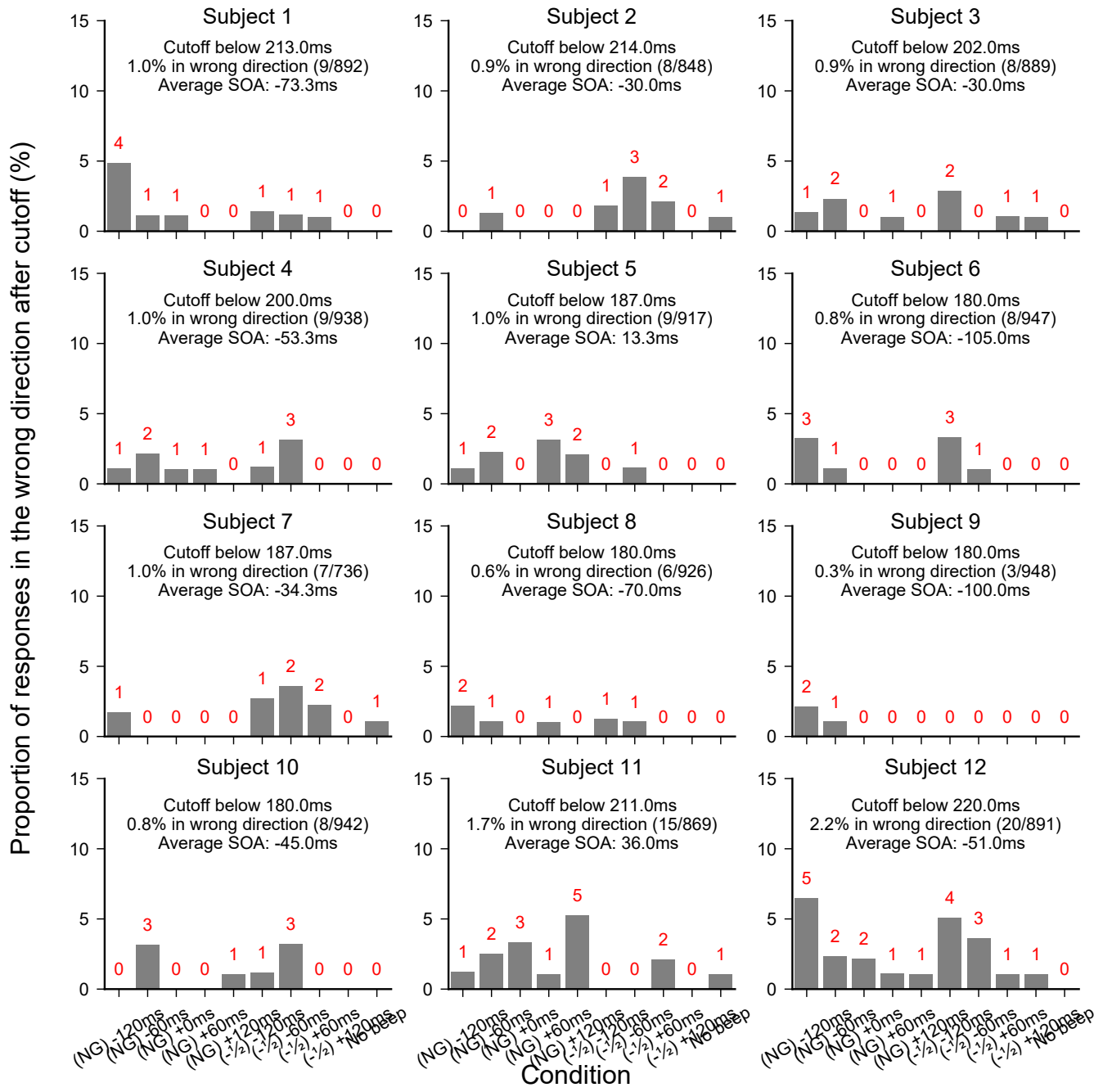

Supplement: S3 Fig — Responses going in the wrong direction of Experiments 1a (left panel) and 1b (right panel). Individual histograms plotting the number of initial responses opposite to where the target appeared for each SOA and No beep conditions. The upper plots shows the initial data with all the correctly detected responses. The lower plots shows the analyzed data after exclusion of anticipative responses by applying the optimal low-pass cutoff filter determined individually (see the Data processing section for further details). (PDF) [file pone.0266468.s005.pdf]

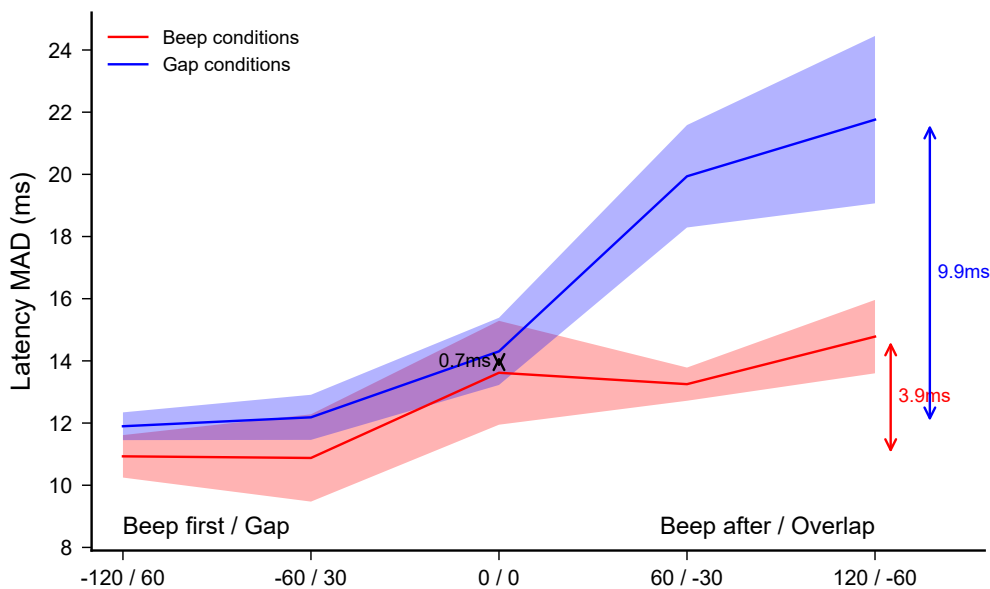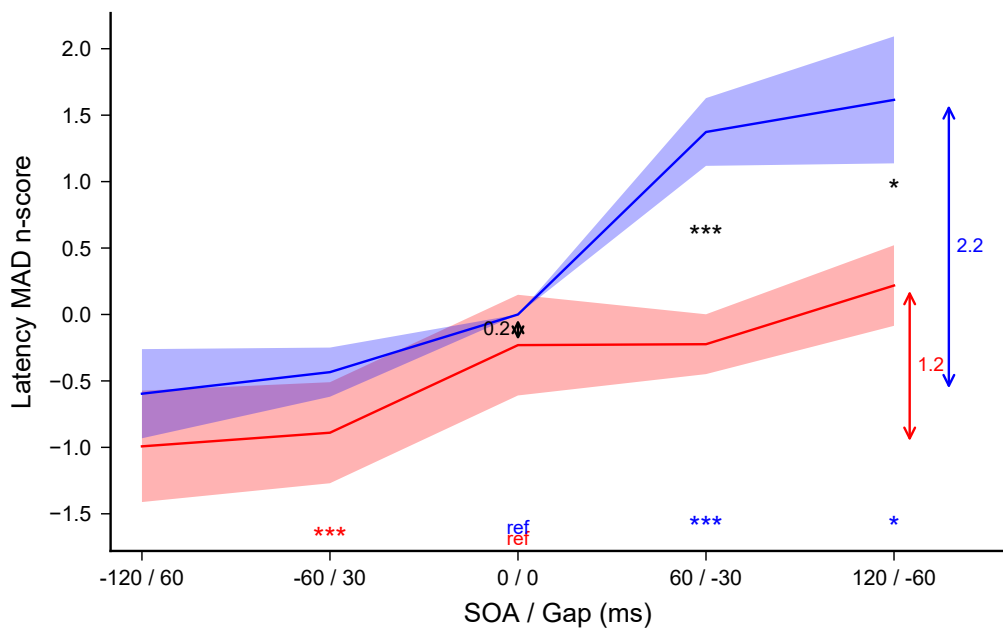

Supplement: S4 Fig — Effect of sound beeps on saccadic RT. Median absolute deviation of the response latency distribution averaged across participants for each SOA condition (top) and their corresponding nscores (bottom). Statistics performed for each group of conditions separately on the nscores, included paired t-tests comparing each SOA or gap condition with the 0-ms SOA or Gap duration reference conditions, respectively (colored stars above the X-axis). Three stars indicate highly significant differences after Bonferroni correction (p<0.0125) while single stars indicate significant differences without correction (p<0.05). Finally, paired t-tests compared Beep and Gap conditions for each SOA (black stars between curves). Three stars indicate highly significant differences for the paired t-tests after Bonferroni correction (p<0.00556). (PDF) [file pone.0266468.s006.pdf]
